# Combining *In Situ* Techniques (XRD, IR, and ^13^C NMR) and Gas Adsorption Measurements Reveals CO_2_-Induced Structural Transitions and High CO_2_/CH_4_ Selectivity for a Flexible Metal–Organic Framework JUK-8

**DOI:** 10.1021/acsami.1c07268

**Published:** 2021-06-08

**Authors:** Kornel Roztocki, Marcus Rauche, Volodymyr Bon, Stefan Kaskel, Eike Brunner, Dariusz Matoga

**Affiliations:** † Faculty of Chemistry, Adam Mickiewicz University, Uniwersytetu Poznańskiego 8, 61-614 Poznań, Poland; ‡ Center for Advanced Technologies, Adam Mickiewicz University, Uniwersytetu Poznańskiego 10, 61-614 Poznań, Poland; § Chair of Bioanalytical Chemistry, Technische Universität Dresden, Bergstrasse 66, 01062 Dresden, Germany; ∥ Chair of Inorganic Chemistry, Technische Universität Dresden, Bergstrasse 66, 01062 Dresden, Germany; ⊥ Faculty of Chemistry, Jagiellonian University, Gronostajowa 2, 30-387 Kraków, Poland

**Keywords:** metal−organic framework, flexibility, *in situ* techniques, adsorption, separation

## Abstract

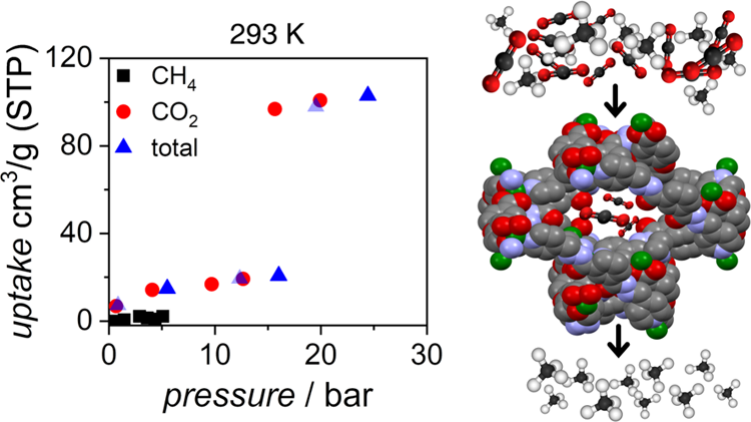

Flexible metal–organic frameworks (MOFs) are promising materials in gas-related technologies. Adjusting the material to processes requires understanding of the flexibility mechanism and its influence on the adsorption properties. Herein, we present the mechanistic understanding of CO_2_-induced pore-opening transitions of the water-stable MOF **JUK-8** ([Zn(oba)(pip)]*_n_*, oba^2–^ = 4,4′-oxybis(benzenedicarboxylate), pip = 4-pyridyl-functionalized benzene-1,3-dicarbohydrazide) as well as its potential applicability in gas purification. Detailed insights into the global structural transformation and subtle local MOF–adsorbate interactions are obtained by three *in situ* techniques (XRD, IR, and ^13^CO_2_-NMR). These results are further supported by single-crystal X-ray diffraction (SC-XRD) analysis of the solvated and guest-free phases. High selectivity toward carbon dioxide derived from the single-gas adsorption experiments of CO_2_ (195 and 298 K), Ar (84 K), O_2_ (90 K)_,_ N_2_ (77 K), and CH_4_ (298 K) is confirmed by high-pressure coadsorption experiments of the CO_2_/CH_4_ (75:25 v/v) mixture at different temperatures (288, 293, and 298 K) and *in situ* NMR studies of the coadsorption of ^13^CO_2_/^13^CH_4_ (50:50 v/v; 195 K).

## 1 Introduction

According to the Intergovernmental Panel on Climate Change (IPCC),^1^ the growing concentration of carbon dioxide in the atmosphere has enhanced the greenhouse effect. This has triggered environmental issues such as droughts, wildfires, flooding, and heatwaves.^2^ Beyond many anthropogenic sources of CO_2_ emission, chemical separation is responsible for the release of 10–15% of global output,^3^ and increasing demand for high-purity chemicals will enhance its impact.

Over 40 years of industrial separation and purification, microporous materials have played a crucial role in these processes,^4,5^*e.g*., zeolites, silica, alumina, and activated carbon are commercially used in paraffins/isoparaffins, N_2_/O_2_, O_2_/N_2_, C_2_H_4_/C_2_H_2_ separation, or CH_4_ purification.^4^ To meet the demand and reduce the impact of the industry on the environment, cooperative efforts are necessary to develop materials and procedures for green technologies.^6^ Recently, a novel class of porous compounds, metal–organic frameworks (MOFs), have emerged as a potential game-changer in gas-related technologies.^7−12^ In 2006, Chen et al. have shown the first gas chromatographic separation of alkanes by using twofold interpenetrated MOF-508.^13^ Since this time, many valuable reports stressed the usefulness of MOFs in purification and separation based on different technologies. For example, Long and co-workers have evaluated rigid MOF-177 and CPO-27 for postcombustion carbon dioxide capture via temperature swing adsorption.^14^ Zaworotko et al. used synergetic MOF sorbents to make ethylene pure enough for producing polymers,^15^ and the Eddaoudi group upgraded natural gas.^16^ Among these reports, it is important to highlight the promising role of MOFs in the construction of semipermeable mixed-matrix membranes for separation processes of industrially important molecules.^17^ Further progress in gas-related technology could result from flexible MOFs, which are a subgroup of metal–organic frameworks that respond to external stimuli like adsorbates with considerable structural transformation.^18−24^ Intrinsic flexibility improves selectivity,^25−27^ considerably increases the working capacity,^28,29^ enables self-accelerating CO sorption,^30^ and influences gas separation.^31^ However, examination of the transition mechanism caused by the external stimulus requires sophisticated *in situ* techniques,^32^*e.g*., nuclear magnetic resonance (NMR),^33^ powder X-ray diffraction (PXRD),^34^ infrared spectroscopy^35^ (IR), and others.^36^ The obtained information serves as input for the understanding–tuning–developing cycle for adjusting the crucial features of the adsorbent for advanced applications.

The acylhydrazone MOFs^37^ are a novel and developing group of metal–organic frameworks that bear the −C(O)=N–NH– motif on the pore surface. Members of this family show very interesting properties, such as extraordinary stability,^38^ structural transformation,^39^ excellent catalytic reactivity in CO_2_ fixation to epoxides,^40^ proton conductivity,^41^ as well as sensing activity.^42^ Reports about guest–framework interactions with similar amide groups can be found in the literature.^43^ However, to the best of our knowledge, there is still lack of comprehensive experimental study concerning the influence of the −C(O)=N–NH– moiety on those MOF properties. In this work, we have bridged this gap by utilization of three complementary *in situ* techniques (IR, PXRD, ^13^C NMR of CO_2_) corroborated with single-crystal X-ray diffraction (SC-XRD). By this approach, we characterize the CO_2_-induced transition mechanism of water-stable **JUK-8** ([Zn(oba)(pip)]*_n_* oba^2–^ = 4,4′-oxybis(benzenedicarboxylate), pip = 4-pyridyl-functionalized benzene-1,3-dicarbohydrazide).^24^*In situ* PXRD provides global information about two-step structural transformation, while *in situ* IR and NMR shed light on the interaction between carbon dioxide and acylhydrazone group (−C(O)=N–NH−).^39 ,40^ Moreover, one-component (CO_2_, CH_4_, Ar, O_2_, and N_2_) and multicomponent (CH_4_/CO_2_) equilibrium adsorption studies in a broad temperature range have shown high selectivity of **JUK-8** toward carbon dioxide.

## 2 Results and Discussion

### 2.1 Guest-Dependent Structural Transformations Elucidated by *Ex Situ* SC-XRD

**Figure 1:**
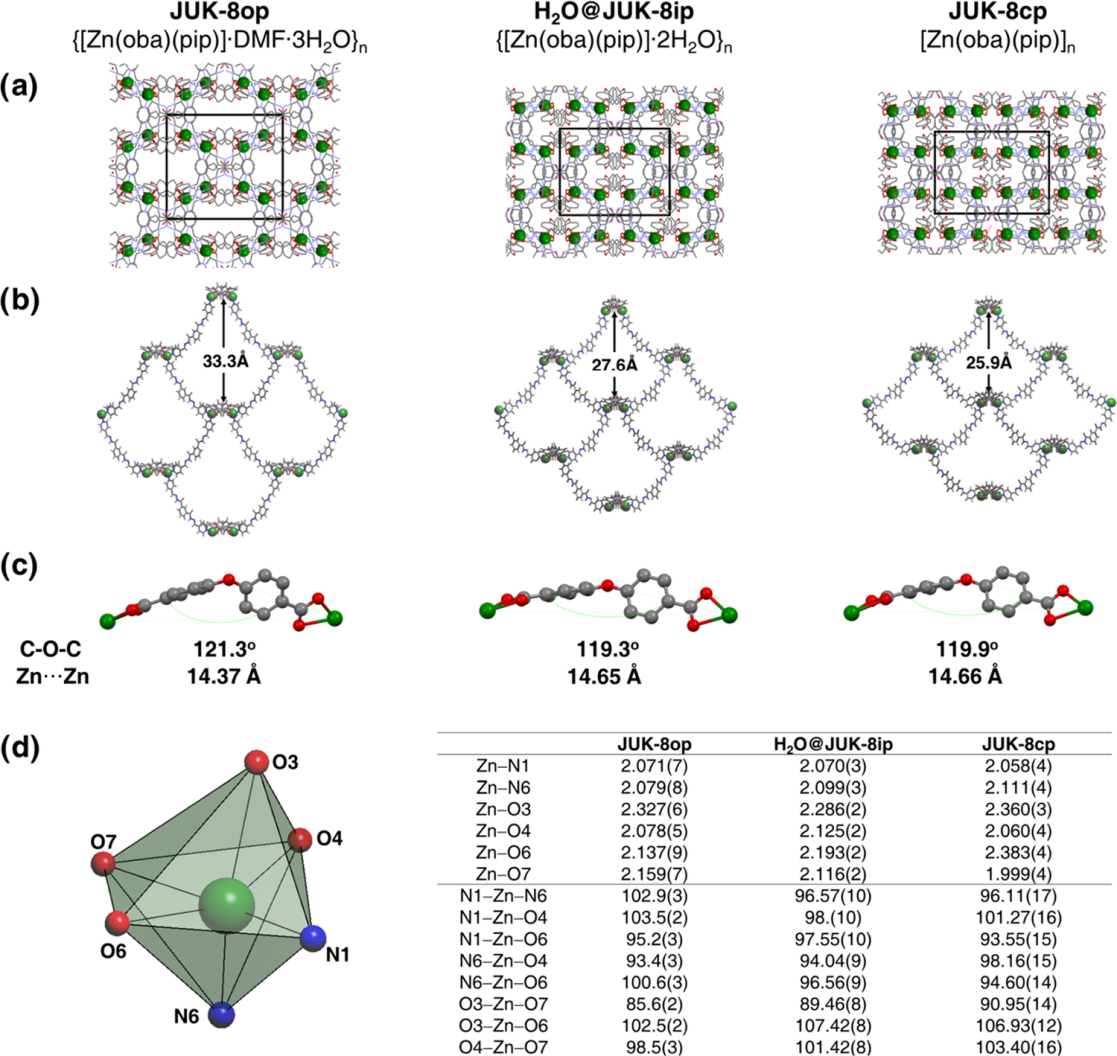
Comparison of three guest-dependent crystalline phases of **JUK-8** elucidated by SC-XRD: (a) packing diagrams showing the entrance to the [001] channel, H atoms are omitted; (b) single subnetworks viewed along the *c* axes; (c) configuration of the oba^2–^ linker, H atoms are omitted; (d) Zn coordination sphere; selected bond lengths (Å) and angles (^o^) in **JUK-8op**, **H**_**2**_**O@JUK-8ip**, and **JUK-8cp** (for more information, see Table S4). Zn, green; C, gray; O, red; N, blue; and H, pale gray.

**JUK-8** (Jagiellonian University in Kraków-8) is a microporous MOF assembled from eight interpenetrated subnetworks held together by hydrogen bonds and π···π stacking interactions (Figures S1 and S2; Tables S1 and S2).^24^ Despite the high level of interpenetration, fully solvated **JUK-8op** {[Zn(oba)(pip)]·DMF·3H_2_O}_n_ (CSD code: ZUFXIK) has one-dimensional zig-zag channels propagating along the [001] direction (Figure 1). Upon thermal removal of guest molecules (443 K, 10^–3^ mbar), further denoted as activation, all eight diamondoid subnetworks collectively breathe to reach a new closed phase **JUK-8cp** ([Zn(oba)(pip)]*_n_*; Figures S3 and S4), whose structure was elucidated in this work by SC-XRD.

Due to the high affinity of **JUK-8cp** toward water, the desolvated phase exposed to a trace amount of moisture immediately transforms to the previously described intermediate phase {[Zn(oba)(pip)]·2H_2_O}*_n_* (**H**_**2**_**O@JUK-8ip**; Figures S3–S5, CSD code: ZUFXOQ).^24^ To prevent water adsorption by **JUK-8cp**, a suitable single crystal of the unknown **cp** phase was placed under an inert atmosphere into a preheated capillary, which was sealed and transferred for synchrotron SC-XRD measurements (Figures S6 and S7).

During the activation of **JUK-8op**, the monoclinic symmetry of the crystal structure (space group **C**2/**c**) remains unchanged; however, the one-dimensional channels transform to zero-dimensional cages (Figure S8). Shrinking of the unit cell volume (8050.1 Å^3^ → 6531 Å^3^) upon transition from the **op** to **cp** phase is accompanied by a considerable contraction of the *a*-axis and moderate changes of *c*, *b*, and β cell parameters (Tables 1 and S3). The meticulous comparison of the three structures reveals that the distances between the nearest symmetry equivalent zinc atoms (Zn···Zn) from different subnetworks are 7.63 Å (**op**), 7.53 Å (**ip**), and 7.43 Å (**cp**). The relatively small difference of Zn···Zn distances (Δ = 0.20 Å) between **op** and **cp** phases proves that the breathing motion practically does not change the relative positions of subnetworks and the observed changes mostly rely on rearrangements around Zn^2+^ cations including slight bending of the oba^2–^ linkers (Figure 1 and Table S4). Thermal removal of guest molecules from **JUK-8op** also causes reinforcement of hydrogen bonds between adjacent subnetworks (N(4)-H(4)···O(3); Table S1), as well as it is responsible for considerable shortening (by 0.25 Å) of the Zn1-O7 bond (Figure 1).

**Figure 2:**
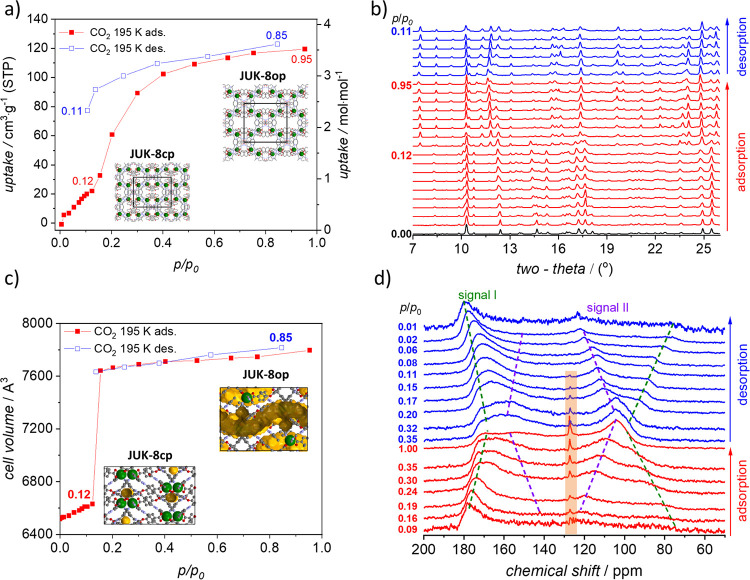
*In situ* monitoring of CO_2_ adsorption by **JUK-8cp** at 195 K: (a) adsorption/desorption isotherms; (b) PXRD patterns (λ = 1.540599 Å) measured in parallel to CO_2_ physisorption; (c) unit cell volume changes during adsorption/desorption; and (d) ^13^C NMR of adsorbed ^13^CO_2_ as a function of pressure increase (adsorption) and subsequent pressure release (desorption) at 195 ± 3 K. Purple and red dashed lines visualize the changes of signals I and II, respectively. A narrow signal at 127.8 ppm of a pure ^13^CO_2_ gas at 1 bar (195 K) is indicated by an orange rectangle.

**Table 1:** Unit Cell Parameters of Investigated Phases Found by SC-XRD and Obtained from PXRD Patterns, Juxtaposed with Corresponding Geometric Porosity Parameters^a^

	*V* /Å^3^	*a* /Å	*b* /Å	*c* /Å	β/deg	space group	*m*_pd_/Å	*p*_ws_/Å	*V*_pt_/cm^3^·g^–1^	*V*_pe_/cm^3^·g^–1^
**JUK-8op^b^**	8050	16.98	18.51	26.16	101.65	monoclinic **C**2/*c*	4.67	4.13	0.241	
**H_2_O@JUK-8ip^b^**	6727	13.87	17.54	27.72	94.34		3.53		0.035	
**JUK-8cp^b^**	6531	12.98	18.06	27.94	94.01		3.43		0.012	
**JUK-8cp^c^**	6518	13.05	17.98	27.84	93.30					
**CO_2_@JUK-8ip^c^**	6631	13.28	18.00	27.82	94.46					0.037
**CO_2_@JUK-8op^c^**	7817	16.04	18.13	26.96	98.87					0.220

a *m*_pd_, maximum pore diameter (Zeo^++^);^44^*p*_ws_, pore window size (Zeo^++^); *V*_pt_, theoretical pore volume data from single-crystal structure (Mercury 4.3.1; probe radius =1.3 Å); and *V*_pe_, experimental pore volume for CO_2_ adsorption (195 K) calculated according to the Gurvich rule (Figure 2).

b Data from single-crystal structure.

c Data from *in situ* powder X-ray diffraction analysis (195 K) at *p*/*p*_0_ = 0.12 and 0.85 for **CO_2_@JUK-8ip** and **CO_2_@JUK-8op**, respectively.

### 2.2 CO_2_-Induced Transformation Monitored by *In Situ* Techniques (PXRD, IR, and ^13^C NMR)

By combining three complementary *in situ* techniques (IR, PXRD, and NMR) during CO_2_ adsorption (195 K), supported by the single-crystal investigation, we shed light on the mechanism of flexibility in **JUK-8** and its influence on the framework properties. PXRD provides global information about CO_2_-induced **JUK-8** breathing (Figure S9), whereas IR and NMR spectroscopies probe interactions between CO_2_ and functional groups.

The measured CO_2_ adsorption isotherm (195 K), followed by *in situ* PXRD, on a ground sample of **JUK-8** demonstrates a good agreement with the *ex situ* data (Figures S10 and S11) and the previously published isotherm.^24^ Unit cell parameters derived from the PXRD pattern of the activated sample match the calculated parameters from the single-crystal structure of the desolvated phase (**JUK-8cp**), and it indicates that the used model is correct (Table 1).

From a structural point of view, the mechanism of CO_2_-induced transition involves two steps. (1) In the first step (pressure range *p* = 0.00–0.12 bar), **JUK-8cp** adsorbs ∼ 0.5 CO_2_ molecules per Zn (**CO**_**2**_**@JUK-8ip**, [Zn(oba)(pip)]·1/2CO_2_) and unit cell volume slightly swells (by 1.7%). It indicates that carbon dioxide molecules occupy 0-D cages, each between two zinc atoms (Figure S8). (2) Exceeding *p* = 0.12 bar causes the second opening step, characterized by the change of pore dimensionality (0-D → 1-D; **CO**_**2**_**@JUK-8ip** → **CO**_**2**_**@JUK-8op**), and the unit cell volume of **JUK-8** abruptly increases to 7640 Å^3^ (by 17.2%). Further adsorption of CO_2_ leads to slight swelling, and the highest unit cell volume is observed at *p* = 0.85 bar on a desorption branch (7812 Å^3^; increase by 19.9%), which is 223 Å^3^ lower than for the H_2_O/DMF-loaded **JUK-8op**.

A comparable mechanism of CO_2_ adsorption is found for the **SNU-9** material.^45^ However, in its desolvated phase, **SNU-9** has one-dimensional channels that enable diffusion. For **JUK-8**, the CO_2_ transport mechanism to 0-D cages is still unknown and will be the subject of further detailed investigations.

**Figure 3:**
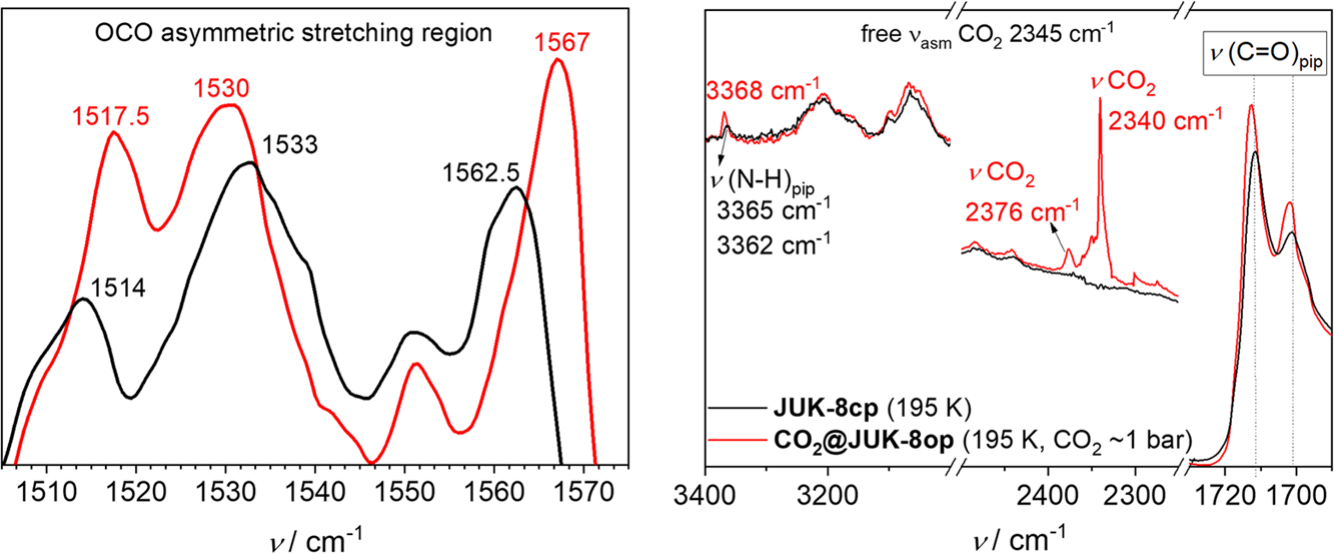
*In situ* IR spectra recorded during carbon dioxide adsorption: OCO asymmetric stretching region for the oba^2–^ carboxylates (left) and characteristic (N–H)_pip_, (C=O)_pip_, and CO_2_ regions (right). Black curve, **JUK-8cp** (195±3 K); red curve, CO_2_-loaded **JUK-8op** (195±3 K; *p*/*p*_0_ = 0.99).

Assuming that the mutual position of the eight subnetworks during CO_2_ adsorption does not change, the transition of **JUK-8cp** to **CO**_**2**_**@JUK-8op**, similarly to the activation process (Figure 1), involves collective breathing of all subnetworks with the rearrangement of zinc coordination spheres (Figure 1 and Table S4). The comparison of the IR spectra collected for **CO**_**2**_**@JUK-8op** under CO_2_-rich atmosphere (∼0.99 bar; 195 K) and for the evacuated sample **JUK-8cp** proves this hypothesis (Figures 3 and S12).

The structural transformation entails alteration of the OCO angles and the Zn–O bond lengths, which is observable by considerable changes in the OCO asymmetric stretching region of the oba^2–^ carboxylate linkers. Additionally, we have observed two signals from the adsorbed CO_2_ (at 2340 and 2376 cm^–1^), as compared to gaseous CO_2_ (2345 cm^–1^). The broad and intense signal at 2340 cm^–1^ can be ascribed to the weakly interacting CO_2_ inside the pore; the second one at a higher wavenumber is associated with a more directional interaction between the framework and carbon dioxide. The meticulous comparison of the bands from acylhydrazone groups (−C(O)=N–NH–; *i.e*., *ν*(N–H)_pip_ and ν(C=O)_pip_) in **JUK-8cp** and **CO**_**2**_**@JUK-8op** shows that electron density is exchanged between CO_2_ and those groups. In the spectra, it is manifested by changes of band position and their intensity. Furthermore, the described observations clearly demonstrate the role of an acylhydrazone in the proposed catalytic mechanism for CO_2_ cycloaddition that leads to terminal/internal epoxides, as described by Suresh and co-workers (Figure S13).^40^

To get a deeper insight into the mechanism of CO_2_ adsorption, we have supported *in situ* IR and PXRD investigations by *in situ* NMR measurements. The latter technique is known for high sensitivity to any changes in the electron structure of the investigated species. It can be used for studying host–guest interactions as well as to distinguish between different adsorbates inside the framework and the nonadsorbed free gas (see, for example, refs (46−53) and references therein). ^13^C NMR spectroscopy of adsorbed CO_2_ is frequently used to characterize porous materials such as MOFs (see, *e.g*., the review articles^53,54,59^). For the investigation of the single- and mixture gas adsorption, the previously reported homebuilt *in situ* apparatus was used.^50^ Pure ^13^CO_2_ gas at 1 bar (195 K) yields a narrow signal at 127.8 ppm in agreement with the literature^51,52^ (Figure 2).

According to *in situ* PXRD, the CO_2_-induced transition (195 K), from **cp** → **CO_2_@ip** → **CO_2_@op** phases, occurs between *p*/*p*_0_ = 0.00 and 0.12 (Figure 2a). However, in the case of *in situ* NMR, possibly due to a minor temperature difference, the gate opening pressure (*gop*) shifts to *p*/*p*_0_ = 0.16–0.19. Furthermore, coexistence of small amounts of different phase impurities visible in *in situ* PXRD collected during CO_2_ adsorption can also have a minor impact (Figures 2a and S11).

In the intermediate phase (**CO**_**2**_**@ip**), the CO_2_ molecules are confined, and their mobility is restricted. Consequently, at *p*/*p*_0_ = 0.09, only a very broad signal (signal I) ranging from ca. 70 to 180 ppm with an intensity maximum at 178 ppm is observed (Figure 2). The line shape is typical for CO_2_ and resembles the line shape observed for signals dominated by chemical shift anisotropy with rotational symmetry in powder samples. The chemical shift tensor then exhibits the two principal values δ_⊥_ (perpendicular to the symmetry axis) and *δ*_||_ (parallel to the symmetry axis). However, the measured chemical shift anisotropy (CSA) Δσ *=* δ_⊥_ – δ_||_ = 110 ppm is considerably lower than the value of 355 ppm, which would be expected for fully immobilized CO_2_ molecules in powder samples.^32,53^ This indicates a restricted mobility in the pores resulting in partial averaging of the CSA. A similar property, the so-called residual dipolar couplings, is a well-known phenomenon in liquid-state NMR spectroscopy.^54,55^ In the case of CO_2_ in MOFs, the molecules rapidly travel through the pores. For spatially anisotropic pore systems, the described averaged line shape results. NMR spectra, SC-XRD data, and *in situ* PXRD analysis indicate that CO_2_ molecules in **CO**_**2**_**@JUK-8ip** are confined in 0-D pores (Figure S8). Notably, the CO_2_ adsorption mechanism corresponds to that previously reported for water vapor;^24^ H_2_O molecules in the **ip** phase are localized in the vicinity of −C(O)=N–NH– and −COO– groups (Figure S5). During stepwise pressure increase (*p*/*p*_0_ = 0.09–0.99), the unit cell parameters rapidly change and the intensity maximum, *i.e*., the principal value δ_⊥_ of signal I (*p*/*p*_0_ =1), shifts from ∼178 to 170 ppm and the effective chemical shift anisotropy Δσ narrows from 110 ppm down to about 80 ppm (Figure 2). Furthermore, at the *gop* of *p*/*p*_0_ = 0.19, an additional signal (signal II) appears. Its effective *CSA* has the opposite sign as signal I. The intensity maximum δ_⊥_ occurs initially at 120 ppm and shifts to lower values at increasing pressures. This second signal becomes more intense during the adsorption, and both signals have comparable intensity at *p*/*p*_0_ = 0.35. In the low-pressure regime (*p*/*p*_0_ = 0.09–0.30), signal II is relatively narrow. At further increasing pressure, the line becomes broader and transforms into the above-described CO_2_ tensor spectrum with an effective Δσ of −70 ppm at *p*/*p*_0_ ∼ 0.99.

To understand subtle local MOF–adsorbate interactions, we also calculated isotropic chemical shift (ICS) for both signals. In the case of signal I (*p*/*p*_0_ = 0.24), an ICS of 144 ppm is determined. This is higher than the value of 127 ppm measured for free bulk gas. The ICS of 121 ppm is obtained for signal II (*p*/*p*_0_ ∼ 0.99), which is close to the value for free gaseous CO_2._^51^ This observation proves the existence of two chemically different CO_2_ states inside the pore system and supports the IR data described above (Figure 3). The considerably increased isotropic chemical shift of the first species of CO_2_ (signal I) compared to that of the second (signal II) indicates that the gas molecules in the MOF exhibit different chemical environments. Taking into consideration the IR, PXRD, and SC-XRD studies, CO_2_ molecules are expected to be adsorbed in the vicinity of acylhydrazone pockets (Figure S5). Two factors have a simultaneous impact on the chemical environment: the pore confinement and amount of adsorbed CO_2_ molecules.

After framework opening, further uptake causes an intensity increase of signal II. These species exhibit weaker CO_2_ interactions with the framework. Increasing gas uptake influences the width of both signals in the opposite direction. Signal I becomes narrower and signal II becomes broader (Figure 2; dashed lines). These data indicate that the mobility of the weaker adsorbed CO_2_ (signal II) is more restricted at high pressure (*p*/*p*_0_ = 0.99), in contrast to that of the strongly interacting species (signal I). This can be explained by the fact that CO_2_ molecules causing signal II inside the partly filled, open pores of **CO**_**2**_**@JUK-8op** are more mobile than at a higher degree of pore filling. On the other hand, higher pressure increases the distance between the adsorbate and functional groups in pockets, enhancing the motional freedom of the confined CO_2_ molecules causing signal I.

In summary, breathing and swelling change the spatial arrangement of the framework, thus considerably influencing the number and mobility of the adsorbed CO_2_ species in **JUK-8**.

**Figure 4:**
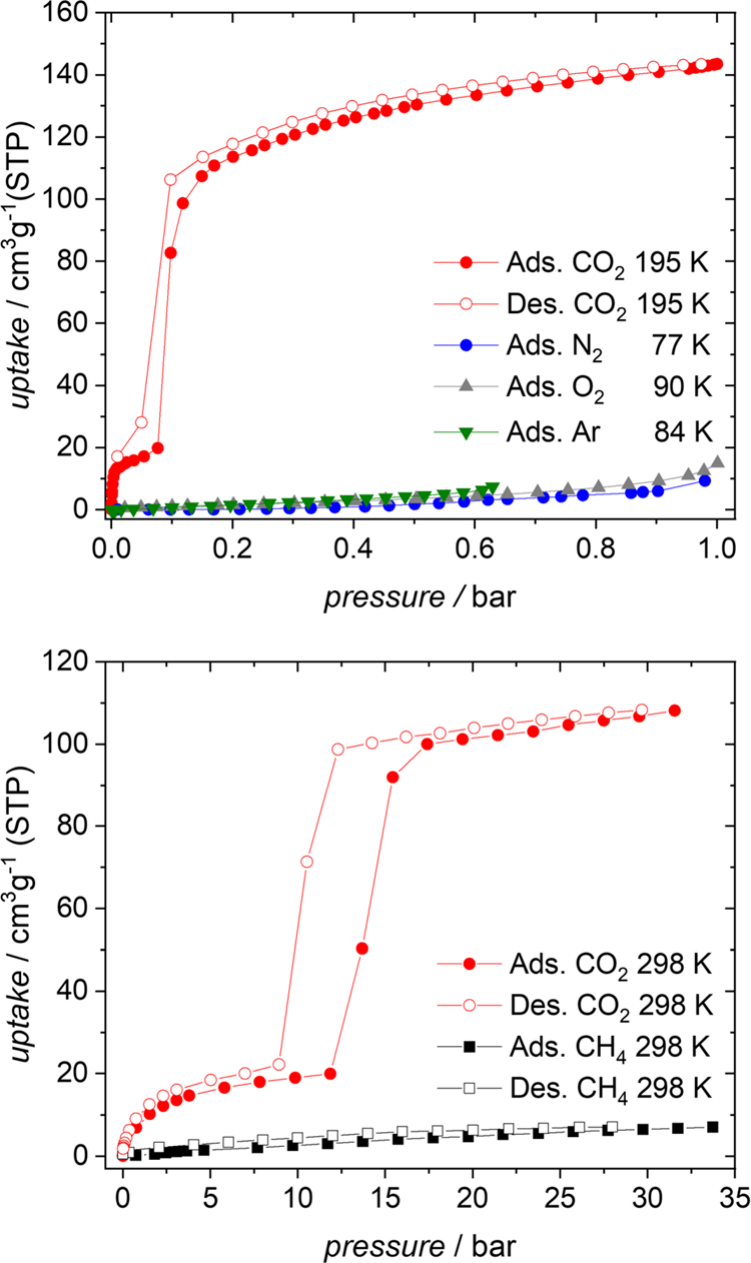
Single-gas adsorption measurements for **JUK-8:** CO_2_ (195 and 298 K; red), Ar (84 K; green), O_2_ (90 K; gray), N_2_ (77 K; blue), and CH_4_ (298 K; black). Full symbols, adsorption and open symbols, desorption.

During desorption, the reverse transition mechanism is observed. However, hysteresis occurs due to MOF···CO_2_ and CO_2_···CO_2_ interactions. Below *p*/*p*_0_ = 0.08, the framework transforms in the **CO**_**2**_**@ip** state (Figure 4); signal I dominates the spectrum and becomes stepwise broader, finally reaching the initial value of 110 ppm at *p*/*p*_0_ = 0.03. Unit cell volume contraction of **JUK-8** during desorption decreases the distance between CO_2_ molecules to −C(O)=N–NH– functional groups and again immobilizes the adsorbate causing the appearance of signal I. The described *in situ* NMR signals are fully reproduced even after 3 cycles, which proves that the CO_2_ environment during the adsorption is independent of the cycling experiment (Figure S14).

### 2.3 Single- and Mixed-Gas Adsorption Properties in a Broad Range of Temperature

Single-gas adsorption isotherms of Ar, N_2_, O_2_, and CO_2_ gases, measured in the low-temperature regime, suggest high selectivity toward carbon dioxide (Figures 4 and S15). Thermally activated MOF, **JUK-8cp**, does not adsorb nitrogen (77 K), argon (84 K), and oxygen (90 K). The total uptake at *p*/*p*_0_ = 0.99 is equal to 6, 6, and 12 cm^3^ for N_2_, Ar, and O_2_, respectively. On the other hand, the activated pristine material adsorbs 144 cm^3^/g CO_2_ (195 K) at 0.99 bar (*p*/*p*_0_ = 0.99) with gate opening pressure at *p* = 0.08 bar (*p*/*p*_0_ = 0.08). Low-temperature adsorption studies indicate the potential applicability of **JUK-8** in gas-related technologies. To assess this, we measured high-pressure single-component CH_4_ and CO_2_ isotherms at an ambient temperature (298 K). Methane, similarly to O_2_, N_2_, and Ar at low temperature, does not open **JUK-8cp**, and the total uptake of CH_4_ at a very high pressure (∼32 bar) is considerably low (7 cm^3^/g) compared to that of CO_2_ at similar conditions (108 cm^3^/g at ∼32 bar and 298 K; Figure 4). On the other hand, due to the different thermodynamic conditions, the absolute value of *gop* shifts from 0.08 bar (195 K, Figure S15) to 11.56 bar (298 K).

**Figure 5:**
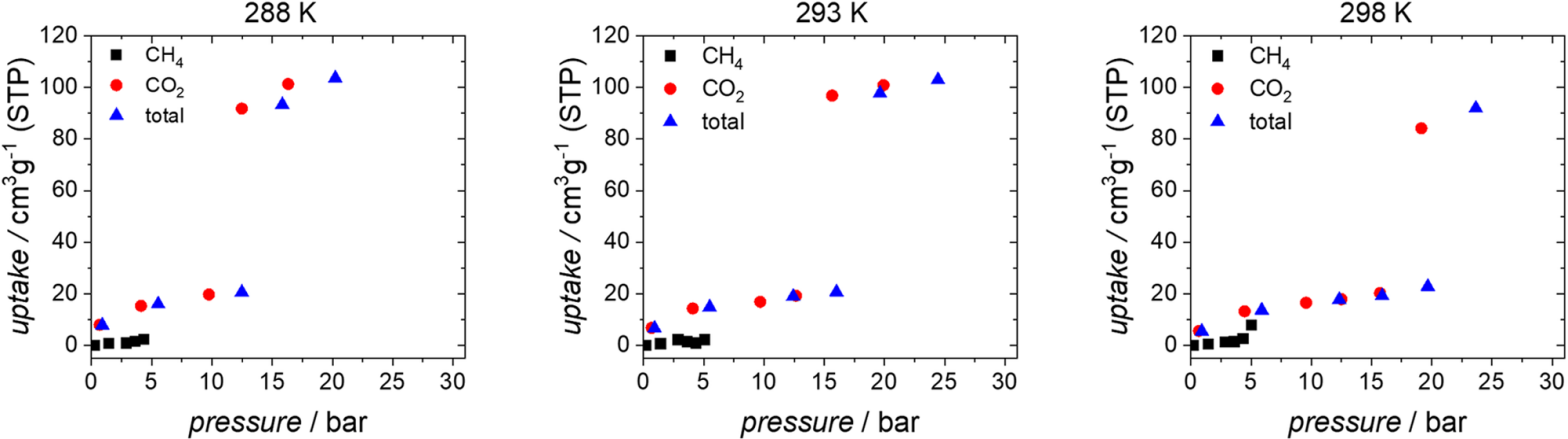
Isothermal multicomponent adsorption experiments for CO_2_/CH_4_ (75:25 v/v) mixtures in **JUK-8** material for three different temperatures. Blue triangles, total adsorbed volume; red circles, partial CO_2_ adsorbed volume; and black squares, partial CH_4_ adsorption.

From a thermodynamic point, the flexible MOFs are not inert to adsorbates and their adsorbing specific areas change during adsorption.^56^ Thus, to prove the preferable adsorption of CO_2_, we used CO_2_/CH_4_ selectivity factor *S* (eq 1) instead of the ideal adsorbed solution theory (IAST), recommended for rigid materials. For the 27.7% content of CH_4_ in a single-component experiment, we have obtained a very good *S* = 17.1 at 298 K (eq 1)
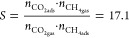
1Here, *n*_CO_2_ads_ and *n*_CH_4_ads_ denote the number of moles adsorbed under the specified gas composition (*n*_CO_2_gas_ and *n*_CH_4_gas_; *p*_CO_2__ =15.42 bar and *p*_CH_4__ = 5.92 bar). It is noteworthy, however, that the calculation based on one-component isotherms may not correspond to real multicomponent adsorption. Recently, a few reports have investigated flexibility during multicomponent experiments;^25,26,57^ however, this type of investigation is still rare and sought after. There are still open questions. Does selectivity for a gas pair arise from the weak affinity of a MOF toward one component? Does the open phase coadsorb a gas that normally does not interact with the closed-pore phase? And what is the influence of temperature? Taking these issues into consideration, we carried out mixed-gas coadsorption experiments for **JUK-8** and CO_2_/CH_4_ (75:25 v/v) gas mixtures at different (288, 293, and 298 K) temperatures (Figure 5).

The narrowest pore diameter of **JUK-8op** is approx. 4.1 Å, which indicates that methane, whose kinetic diameter is 3.8 Å, could be coadsorbed. However, regardless of temperature, CH_4_ does not enter one-dimensional channels and at *p*_CH_4__ ∼ 5 bar, **JUK-8** adsorbs only 3–7 cm^3^ of methane. Furthermore, *S* values calculated from coadsorption of the CO_2_/CH_4_ (75:25 v/v) at ∼20 bar are equal to 8.83, 9.48, and 1.99 for 288, 293, and 298 K, respectively (Figure S16); the obtained values are consistent with one-component isotherms for CH_4_ and CO_2_ at 298 K (*S =* 17.1). This indicates that **JUK-8** shows selectivity factor higher than zeolites and activated carbon and is comparable to MIL-125 and its derivatives (Table S5).

Pure CO_2_ opens the framework at *p* ≈ 11.56 bar (298 K), while the presence of CH_4_ considerably increases *gop* to 15.70 bar (298 K). This effect is caused by gas competition. In the studied temperature range, we have observed a strong linear relationship (*p*_gop_ =0.59–160T; *R*^2^ = 0.999) between *gop* and temperature (Figure S17). In the methane-rich atmosphere, CO_2_ opens the framework at 9.75, 12.66, and 15.70 bar for 288, 293, and 298 K, respectively. On average, each increase of temperature by 5 K causes the increase of *gop* by 3 bar. Furthermore, independent of temperature and type of experiment, the amount of adsorbed CO_2_ before *gop* is almost constant (18–22 cm^3^·g^–1^; [Zn(oba)(pip)]·1/2CO_2_), which indicates that the mechanism of the transition does not depend on the studied conditions.

**Figure 6:**
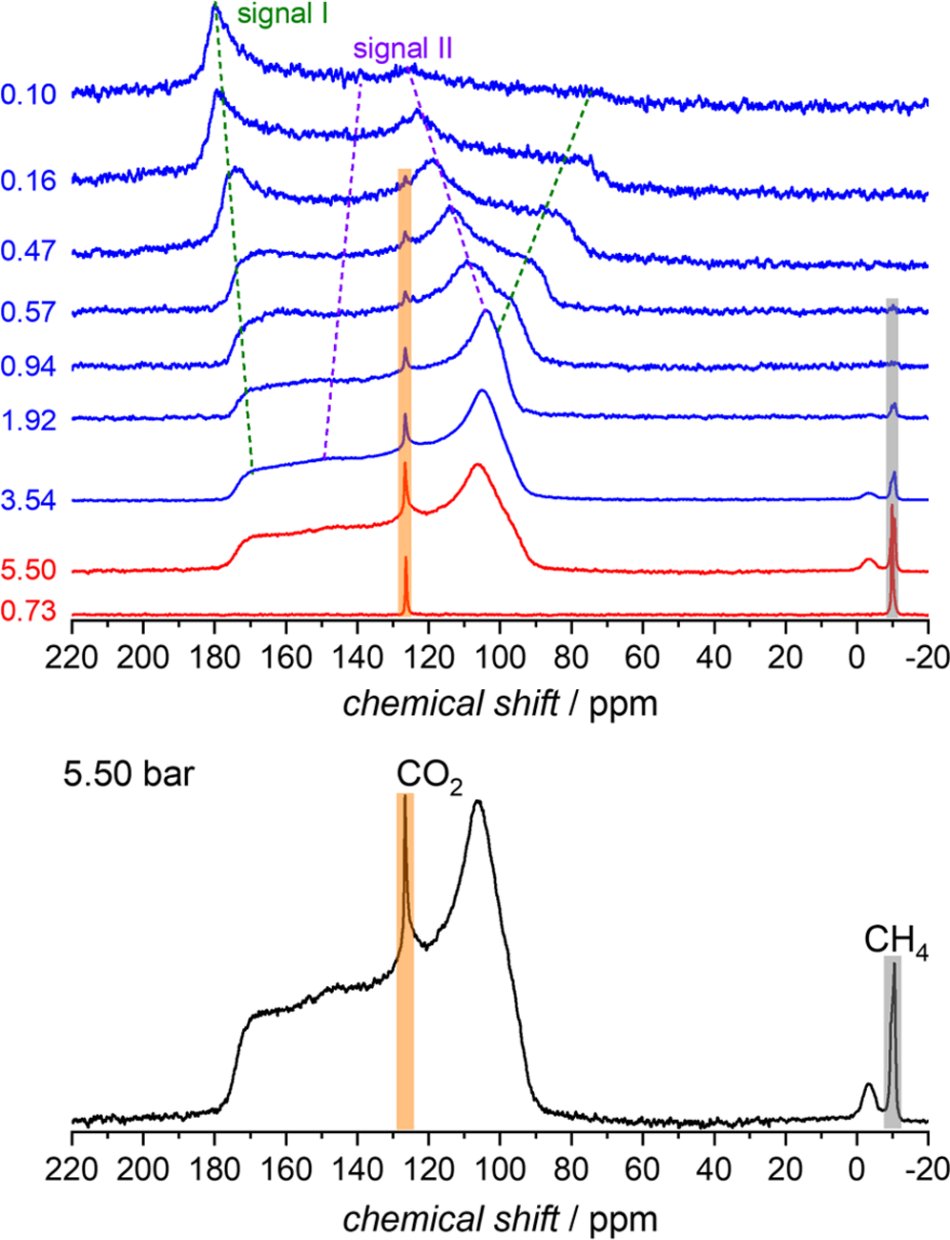
*In situ*^13^C NMR spectra of **JUK-8** during adsorption of the gas mixture (CO_2_ and CH_4_, molar ratio 1:1, 195 K) at selected pressures (top) and ^13^C spectrum of the ^13^CO_2_/^13^CH_4_ gas mixture at 5.50 bar (bottom). The signal at −10 ppm (gray bar) corresponds to gaseous methane, at −4 ppm to the adsorbed methane, at 127 ppm to the gaseous carbon dioxide (orange bar), and the broad signal II between 90 and 180 ppm to the adsorbed carbon dioxide. Signal II gradually transforms into the even broader signal I during pressure release, as can be seen in the spectra shown on top as well as in Figure 2.

To characterize the selectivity in the low-temperature regime, we performed an *in situ*^13^C NMR coadsorption experiment using a ^13^CO_2_/^13^CH_4_ mixture (molar ratio 1:1) at 195 K (Figures 6 and S18). At a total pressure of 0.73 bar, we observed two narrow gas-phase signals resulting from gaseous CH_4_ and CO_2_ at approx. −10 and 127 ppm, respectively.^58,59^ There is no evidence for adsorbed CH_4_ at this pressure. However, the gas-phase signal of CO_2_ is weaker than that of CH_4_, which indicates the presence of adsorbed CO_2_. However, the broad signals of adsorbed CO_2_ are not detectable at such low pressures. After stepwise increasing the total pressure, signal II of adsorbed CO_2_ with an effective *CSA* of ca. −70 ppm finally dominates the spectrum in analogy to the single-component adsorption experiment described above. Since the CO_2_ signal shapes are not significantly different from the single-phase adsorption studies, it is concluded that the adsorption and switching mechanism are not significantly influenced by the presence of CH_4_. At a maximum pressure of 5.50 bar, a relatively weak signal at −4 ppm becomes detectable, which is caused by the adsorbed methane. It means that only minor amounts of methane coadsorb on **JUK-8op@CO**_**2**_ even at low temperature and high pressure. The intensity of the signal due to adsorbed methane only corresponds to 0.6% of the signal II of adsorbed CO_2_. This is 1 order of magnitude less than the amount measured by mixed-gas coadsorption experiments (2.2–8.3%). The selectivity factor (*n*_CH_4_gas_·*n*_CO_2_ads_)/(*n*_CH_4_ads_·*n*_CO_2_gas_) calculated from these *in situ* NMR data at 5.50 bar amounts to 160. During pressure release, the MOF first releases CH_4_. At 1.92 bar, no adsorbed methane and only small amounts of free gas are observable. Further pressure reduction results in a decreasing intensity of the signals of free and adsorbed CO_2_ molecules and the MOF switches back into the intermediate phase.

The selectivity factor of 160 calculated from ^13^C *in situ* NMR data during adsorption is considerably higher than the value obtained from single- and multicomponent volumetric adsorption measurements (1.99–17.1). The observed difference can be explained by the following aspects influencing eq 1. (i) The maximum uptake of CO_2_ at 298 K (108 cm^3^/g at STP) is considerably lower than at 195 K (144 cm^3^/g at STP), *i.e*., *n*CO_2ads_ at 195 K is higher compared to those at 288, 293, and 298 K (Figure 4 and 5). (ii) The accuracy of the *in situ* NMR, compared to the volumetric experiment, is higher, thus enabling a lower detection limit for adsorbed methane. In the NMR experiments, we detected very low amounts of the labeled methane (*n*_CH_4_ads_), as can be seen in Figure 6. (iii) The initial CO_2_/CH_4_ ratio, equal to 1:1 (50:50 v/v) in the ^13^C NMR study, is different from 3:1 (75:25 v/v) in the multicomponent volumetric studies.

### 2.4 Stability in Water

Apart from a structural flexibility, a promising adsorbent for gas-related technologies has to meet other criteria such as stability to impurities,^60,61^ selectivity, endurance for repeatable adsorption–desorption stress,^62^ and lack of adsorption hysteresis. In the previous work, it has been shown that **JUK-8** is chemically stable, *e.g*., immersed in water for 24 h does not change its adsorption properties (shape of isotherm and total capacity) and withstands repeatable vapor adsorption/desorption.^24^ Herein, we repeatedly (hundred times) immersed **JUK-8** in water that was subsequently evaporated by heating at a lowered pressure. We monitored the process by powder X-ray diffraction and infrared spectroscopy, and both techniques provided evidence that the structure of **JUK-8** remains intact (Figure S19). In summary, **JUK-8** is very stable, highly selective toward CO_2_, and resistant to repeatable adsorption/desorption stress. However, it has disadvantages such as high gate opening pressure at a working temperature, moderate CO_2_ uptake, and hysteresis.

## 3 Conclusions

In this work, we have determined the crystal structure of a desolvated closed-pore phase (**JUK-8cp**) of a flexible water-stable metal–organic framework **JUK-8**, which enables a detailed insight into the mechanism of its phase transitions caused by thermal and pressure stimuli. The removal of solvent molecules from **JUK-8** practically does not change the relative positions of its component subnetworks, whereas the structural transition involves rearrangements around zinc cations with slight bending of the carboxylate linkers. The structural analysis carried out by *in situ* PXRD during the adsorption of CO_2_ indirectly indicates the existence of two minima in the free-energy profile of the investigated MOF, while *in situ* IR and NMR spectroscopies uncover preferential positions of the adsorbed CO_2_ molecules. The detailed analysis of one- and multicomponent equilibrium adsorptions at a broad temperature range demonstrates that **JUK-8cp** is a highly selective adsorbent of CO_2_ from CO_2_/CH_4_ mixtures. In summary, this work provides versatile insights toward the understanding of adsorbate–flexible MOF interactions, which is essential for further development of high-performance materials that could meet the expectations of energy-efficient industry.

## 4 Experimental Section

### 4.1 Synthesis

**JUK-8** and 4-pyridyl-functionalized benzene-1,3-dicarbohydrazide (pip) were prepared according to the published method.^24^ All other reagents and solvents were of analytical grade (Sigma-Aldrich, POCH, Polmos) and were used without further purification.

### 4.2 Single-Crystal X-ray Diffraction

Due to high affinity of **JUK-8cp** toward water,^24^ a suitably sized single crystal of desolvated **JUK-8cp** was picked up in a glovebox (MBRAUN) equipped with the Leica microscope (Figure S6). The crystal was closed in a priori activated (453 K for ∼2 h) borosilicate glass capillary (*d* = 0.3 mm). The data set was collected at the BESSY MX BL14.3 beamline of Helmholtz-Zentrum Berlin für Materialien und Energie.^63^ Monochromatic X-ray radiation with a wavelength of *λ* = 0.8939 Å (*E* = 13870 eV) was used in experiments. The data set was collected at 100 K. The crystal symmetry and scan range were determined in each particular case using the iMosflm program.^64^ The φ-scans with an oscillation range of 1° were used for data collection. For each data set, 180 images were collected to reach the maximal possible completeness. The data set was processed in the automatic regime using XDSAPP 2.0 software.^65^ The crystal structure was solved by direct methods and refined by full-matrix least squares on *F*^2^ using the SHELX-2018/3 program package.^66^ All nonhydrogen atoms were refined in anisotropic approximation. Hydrogen atoms were refined in geometrically calculated positions using “riding model” with *U*_iso_(H) = 1.2*U*_iso_(C). CCDC2072669 contains the supplementary crystallographic data for **JUK-8cp**. Experimental data on single-crystal X-ray experiments are summarized in Table S1.

### 4.3 IR Spectra

IR spectra were recorded on a Thermo Scientific Nicolet iS10 FT-IR spectrophotometer equipped with an iD7 diamond ATR attachment.

### 4.4 ***In Situ*****IR**

*In situ* IR spectra were recorded on a Bruker Tensor 27 spectrometer equipped with an MCT detector and working with a spectral resolution of 2 cm^–1^. Prior to the adsorption of CO_2_ at 195 K (Linde Gas Polska, 99.95% used without further purification), the samples were ground and activated in the form of self-supporting wafers for 2 h at 473 K.

### 4.5 Powder X-ray Diffraction

PXRD patterns were recorded at room temperature (295 K) on a Rigaku Miniflex 600 diffractometer with Cu Kα radiation (*λ* = 1.5418 Å) in a 2*θ* range from 3 to 45° with a 0.05° step at a scan speed of 2.5° min^–1^.

### 4.6 *In Situ* Powder X-ray Diffraction

PXRD patterns during the CO_2_ adsorption were measured at Helmholtz-Zentrum Berlin für Materialien und Energie on KMC-2 beamline. The detailed description of the measuring setup is provided in the literature.^34^ Prior to experiments, the as-synthesized **JUK-8op** was ground (∼5 min in a mortar and pestle) and evacuated at 443 K for ∼16 h. For the part of **JUK-8cp** prepared in this way, a CO_2_ isotherm at 195 K was recorded (Figure S10). Due to the heterogeneous distribution of crystallographic orientations (texture) of a polycrystalline material, the degassed **JUK-8cp** was again ground before *in situ* experiments. PXRD patterns, measured during the adsorption and desorption of CO_2_ at 195 K, were indexed using the DICVOL program, integrated into the FullProf.2k V.6.30. Further, the Le Bail fit was performed to refine the unit cell and profile parameters (Figure S11).

### 4.7 *In Situ* NMR

^13^C NMR measurements of ^13^C-enriched CO_2_ and CH_4_ were carried out using a BRUKER Avance 300 spectrometer at 195 K combined with a homemade *in situ* high-pressure gas adsorption apparatus. The apparatus is equipped with a gas mixing chamber to produce the desired CO_2_/CH_4_ gas mixture with a molecular ratio of 1:1 (Figure S18). It was adjusted by first filling the chamber with ^13^CH_4_ up to a certain pressure and afterward adding ^13^CO_2_ up to the final pressure. The pressures were always well below the critical pressure for both gases. This allows us to consider the gases as ideal, *i.e*., the gas pressure is assumed to be directly proportional to the gas concentration/number density of the molecules. Temperature calibration was carried out using the well-known temperature dependence of the ^1^H NMR signal of methanol.^67^ The solvent-free samples were transferred into a 5 mm single-crystal sapphire tube in an argon-filled glovebox. The sample was activated again in the tube under high vacuum for 2 h before the measurements. After pressurization, the samples were equilibrated at least for 30 min to reach thermal equilibrium. The pressure was incremented stepwise by adding the required portion of the initial gas mixture to the sample tube. To ensure equilibrium state after each pressure increase, a 15 min equilibrium phase was allowed. The ^13^C NMR spectra were recorded at a resonance frequency of 75.47 MHz under ^1^H-decoupling using a 10 mm double resonance probe head, a 10.7 μs pulse length for ^13^C, and with a relaxation delay of 5 s. The chemical shifts were referenced relative to ethylbenzene. For rigid CO_2_, the chemical shift anisotropy (CSA) tensor has an overall width of 335 ppm. To ensure excitation of the full width of this, a sufficiently short pulse length must be chosen. We decided to choose a pulse flip angle of 60° to avoid the excitation problem and to decrease the relaxation delay to only 4 times *T*_1_ for quantitative measurements. Under our conditions, *T*_1_ values below 1 s are observed in full agreement with the literature.^67−70^ The chosen recycle delay of 5 s is thus safely longer than 5 times of *T*_1_ and ensures full relaxation of the spin system, *i.e*., saturation effects can be excluded. Moreover, the comparison of the ^13^C NMR spectra with and without ^1^H-decoupling shows that NOE effects have only negligible influence upon the signal intensities under the applied conditions (Figure S18).

### 4.8 Single-Component Gas Adsorption Measurements

Prior to the physisorption measurements, the as-synthesized **JUK-8** was evacuated at 443–453 K for ∼16 h. The Ar physisorption experiment was conducted with an AUTOSORB-iQ-C-XR from Quantachrome at 87 K (cryostat). Nitrogen (77 K), carbon dioxide (195 K), and oxygen (90 K) adsorption/desorption studies were performed on a BELSORP-max adsorption apparatus (MicrotracBEL Corp.), connected to the closed cycle helium cryostat DE-202AG (ARS). The adsorption temperature was set by a temperature controller LS-336 (LAKE SHORE), and the heat produced by the cryostat was removed from the system by a water-cooled helium compressor ARS-2HW. The sample was placed in a custom-made cell consisting of a 3 cm long rod-shaped copper cell of 1 cm diameter, sealed by a copper gasket from the exterior with a copper dome and insulated by dynamic vacuum (*p* < 10^–4^ kPa), and connected to the BELSORP-max adsorption instrument with a 1/8 inch stainless steel capillary.

### 4.9 High-Pressure Single-Gas Adsorption and Mixed-Gas Coadsorption Experiments

Volumetric high-pressure single-gas and mixed-gas adsorption experiments were conducted using the BELSORP-VC (Microtrac MRB) instrument. Helium gas (99.999% purity) was used for the dead volume measurement. Carbon dioxide (99.999% purity) and methane (99.999% purity) gases were used in adsorption experiments. All gases were purchased from Praxair.

All isotherms were measured on the same sample (*m* = 0.6321 g). The sample was degassed in dynamic vacuum over 24 h at 463 K in the measurement cell. Single-gas adsorption isotherms were measured at 298 K in a pressure range of 52–4262 kPa for CH_4_ and 0.5–4207 kPa for CO_2_. Mixed-gas adsorption was measured using the gas mixture of 75% CO_2_ and 25% CH_4_ (v/v) at 298, 293, and 288 K. The gas mixture composition and adsorption temperatures were chosen because of the pressure limitation of the instrument for gas mixtures. The gas mixture was prepared directly in the standard volume part of the instrument from the pure gases for each point of the isotherm separately. After dynamic mixing of the gases for 60 min, the composition of the mixture was determined by the gas chromatograph Agilent 490 Micro-GC-System (GC), coupled to the instrument manifold. The gas mixture was further purged through the sample cell over 60 min, and the overall adsorbed amount was determined from the pressure drop, taking the nonideality correction for each mixture component into account. The composition of the gas mixture after adsorption was analyzed by GC. To increase the reproducibility of the measurements, five GC measurements were done before and after adsorption. The adsorbed amount of mixture components was calculated from the difference in the mixture composition before and after adsorption. Before the measurement of each adsorption point, the sample was degassed in the ultrahigh vacuum for 60 min at 298 K. For each temperature, 5–6 points were measured reaching the maximal equilibrium pressure for the gas mixture of 2366 kPa at 288 K, 2443.4 kPa at 293 K, and 3000 kPa at 288 K.

### 4.10 Stability in Water

**JUK-8op** (*m* = 200 mg) was repeatedly (100 times) immersed in approx. 1.2 mL of distilled water followed by evaporation at 393 K and 450 mbar. After a few evaporation cycles, IR spectra and/or PXRD patterns of the residue were recorded (Figure S19).

## Supporting Information Available

Additional experimental data and structural visualizations (PDF)
X-ray crystal structures: CCDC2072669 (**JUK-8cp**) (CIF)
X-ray crystal structures: CCDC1833889 (**JUK-8op**) (CIF)
X-ray crystal structures: CCDC1875148 (**H_2_O@JUK-8ip**) (CIF)

## Supplementary Material

am1c07268_si_001.pdf

am1c07268_si_002.cif

am1c07268_si_003.cif

am1c07268_si_004.cif
