# Supplementary material for: Combining In Situ Techniques (XRD, IR, and 13C NMR) and Gas Adsorption Measurements Reveals CO2-Induced Structural Transitions and High CO2/CH4 Selectivity for a Flexible Metal–Organic Framework JUK-8
Source: ACS Appl Mater Interfaces. 2021 Jun 8;13(24):28503–13. doi: 10.1021/acsami.1c07268 (PMC8289234; doi:10.1021/acsami.1c07268)
Supplement: Supplementary file 1 — am1c07268_si_001.pdf [file am1c07268_si_001.pdf]

Supporting Information

Combining *in situ* techniques (XRD, IR and ^{13}C-NMR) and gas adsorption measurements reveals CO_2 induced structural transitions and high CO_2/CH_4 selectivity for a flexible metal-organic framework JUK-8

Kornel Roztocki[a,b], Marcus Rauche[c], Volodymyr Bon[d], Stefan Kaskel[d], Eike Brunner[c], and Dariusz Matoga[e]**

[a] Faculty of Chemistry, Adam Mickiewicz University, Uniwersytetu Poznańskiego 8, 61-614 Poznań, Poland

[b] Center for Advanced Technologies, Adam Mickiewicz University, Uniwersytetu Poznańskiego 10, 61-614 Poznań, Poland

[c] Chair of Bioanalytical Chemistry, Technische Universität Dresden, Bergstrasse 66, 01062 Dresden, Germany

[d] Chair of Inorganic Chemistry, Technische Universität Dresden, Bergstrasse 66, 01062 Dresden, Germany

[e] Faculty of Chemistry, Jagiellonian University, Gronostajowa 2, 30-387 Kraków, Poland

Corresponding Author

kornel.roztocki@amu.edu.pl
dariusz.matoga@uj.edu.pl

Table of Contents

Figure S1 Synthetic route to **JUK-8op** including: organic building blocks; representation of an adamantoid cage in a single network (Zn-green, C-grey, O-red, N -blue and H atoms are omitted); eightfold interpenetrated networks viewed along [100]. 3

Figure S2 Crystal structure of **JUK-8op**: N-H···O hydrogen bonds and π···π interactions between adjacent subnetworks. (Zn-green, C-grey, O-red, N -blue and H atoms are omitted) 3

Figure S3 Comparison of three guest-dependent crystalline phases of **JUK-8** elucidated by SC-XRD: crystal structures of the as-made **JUK-8op**[1], **H_2O@JUK-8ip**[1] and **JUK-8cp** (Zn-green, C-grey, O-red, N -blue, H atoms are omitted). 4

Figure S4 Partial overlapping of crystal structures of the as-synthesized **JUK-8op** (blue), **H_2O@JUK-8ip** (purple) and **JUK-8cp** (orange). 5

Figure S5 a) H_2O isotherm (298 K) for **JUK-8cp**. b) Hydrogen bonds (cyan lines) involving three independent subnetworks and water molecules. 5

Figure S6 Glovebox with an inert atmosphere (Ar) equipped with Leica microscope. 6

Figure S7 Optical microscopic images of **JUK-8op**, **H_2O@JUK-8ip** and **JUK-8cp** (closed in the borosilicate capillary). 6

Figure S8 Structural transformation of **JUK-8** induced by desolvation: (Top) Voids calculated using the contact surface: **JUK-8op** (probe radius 1.75 Å)**;** **H_2O@JUK-8ip** and **JUK-8cp** (probe radius 1.3 Å); view along the a axis. (Bottom) Pore size distribution calculated[2] using Zeo^{++} for **JUK-8cp** and **JUK-8op**. 7

Figure S9 Structural transformation induced by CO_2 after exceeding gate opening pressure ($p/p_0 = 0.08$; 0.08 bar). 8
Figure S10 CO_2 isotherm (195 K) for a ground sample of **JUK-8**. 8
Figure S11 a) CO_2 (195 K) adsorption isotherm for **JUK-8cp**. b), c) Corresponding changes of unit cell parameters, and d) in situ variable pressure PXRD (λ = 1.54056 Å) during CO_2 adsorption and desorption. Full symbols - adsorption, open symbols – desorption. 9
Figure S12 In situ IR spectra recorded during carbon dioxide adsorption. Black curve **- JUK-8cp** (195 K), red curve **-** CO_2 loaded **JUK-8op** (195 K; $p/p_0 = 0.99$). 10
Figure S13 Proposed catalytic mechanism catalytic mechanism for CO_2 cycloaddition[3] that leads to terminal / internal epoxides using acylhydrazone MOF {[Co(OBA)(L)]·xG}n (**CoMOF-1**) (H_2OBA = 4,4′-Oxybis(benzoic acid); L = (E)-N'-(pyridin-4-ylmethylene) isonicotinohydrazide; G = DMF, EtOH, MeOH, H_2O) as heterogeneous catalyst. (Reproduced with permission from RSC)[3] 11
Figure S14 In situ $^{13}CO_2$ NMR cycling experiment. 11
Figure S15 Adsorption properties of **JUK-8**: Single component isotherms for CO_2 (195 K), N_2 (77 K), O_2 (90K) and Ar (84 K). 12
Figure S16 Pressure dependence of the selectivity factor S at 288 K, 293 K and 298 K. 13
Figure S17 Correlation between CO_2 induced gate opening pressure (gop) and the temperature observed in the mixed gas adsorption. Gas mixture of 75 % CO_2 and 25 % CH_4 (v/v) at 298 K, 293 K and 288 K. 13
Figure S18 In situ ^{13}C NMR spectra of the pure of $^{13}CO_2/^{13}CH_4$ (1 : 1) gas mixture measured at 1.00 bar without (left) and with (right) ^1H decoupling. Note that the signal intensities (integral areas) for both measurements are very close to the expected 1 : 1 ratio. 14
Figure S19 PXRD patterns (left) and IR spectra monitoring stability of **JUK-8** during the repeatable water solvation and desolvation processes. 14
Figure S20 Comparison of PXRD patterns for **JUK-8op:** the calculated, based on SC-XRD measurement at 120 K (black) and the experimental, measured for the as-synthesized polycrystalline sample at 295 K (blue). 15

Table S1 Hydrogen bond parameters for **JUK-8op, H_2O@JUK-8ip** and **JUK-8cp** (bond lengths Å, and angles in °). 15
Table S2 $\pi\cdots\pi$ interactions for **JUK-8op, H_2O@JUK-8ip** and **JUK-8cp** (bond lengths in Å, and angles in °). 16
Table S3 Crystallographic data for **JUK-8op, H_2O@JUK-8ip** and **JUK-8cp**. 16
Table S4 Selected bond lengths (Å) and angles (°) for **JUK-8op, H_2O@JUK-8ip** and **JUK-8cp**. 17
Table S5 Selectivity factor (S) of CO_2/CH_4 for various MOFs, zeolites and activated carbon. 18

Figures

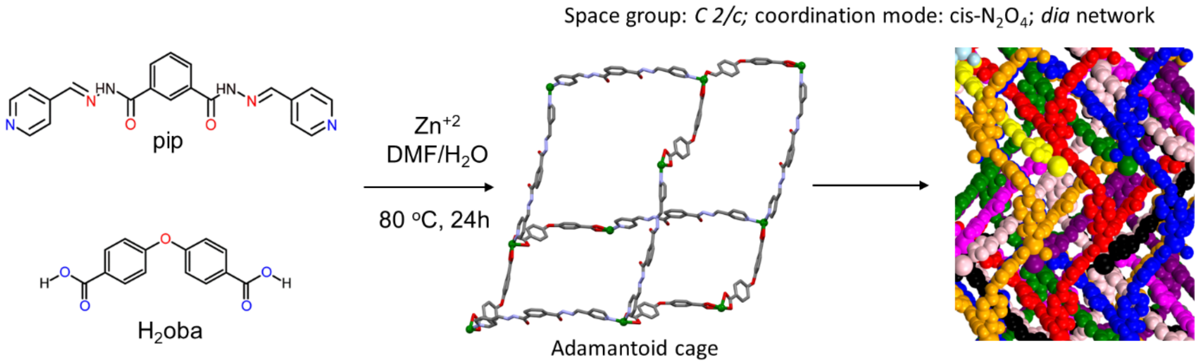

Figure S1 Synthetic route to **JUK-8op** including: organic building blocks; representation of an adamantoid cage in a single network (Zn-green, C-grey, O-red, N -blue and H atoms are omitted); eightfold interpenetrated networks viewed along [100].

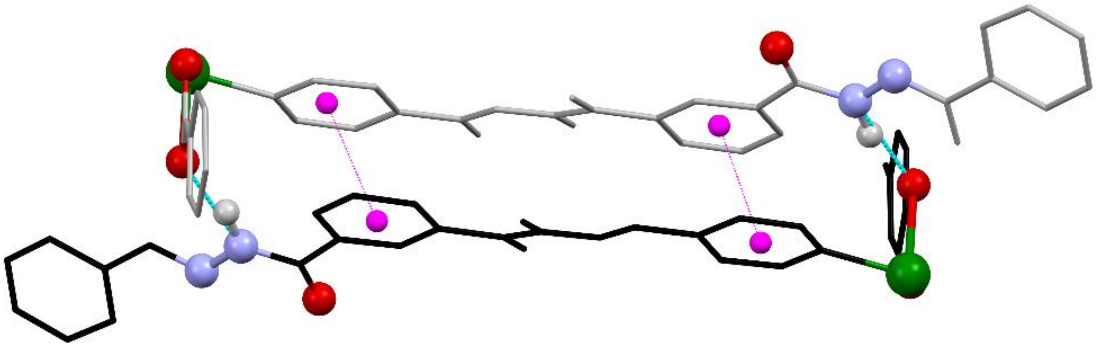

Figure S2 Crystal structure of **JUK-8op**: N-H···O hydrogen bonds and π···π interactions between adjacent subnetworks. (Zn-green, C-grey, O-red, N -blue and H atoms are omitted).

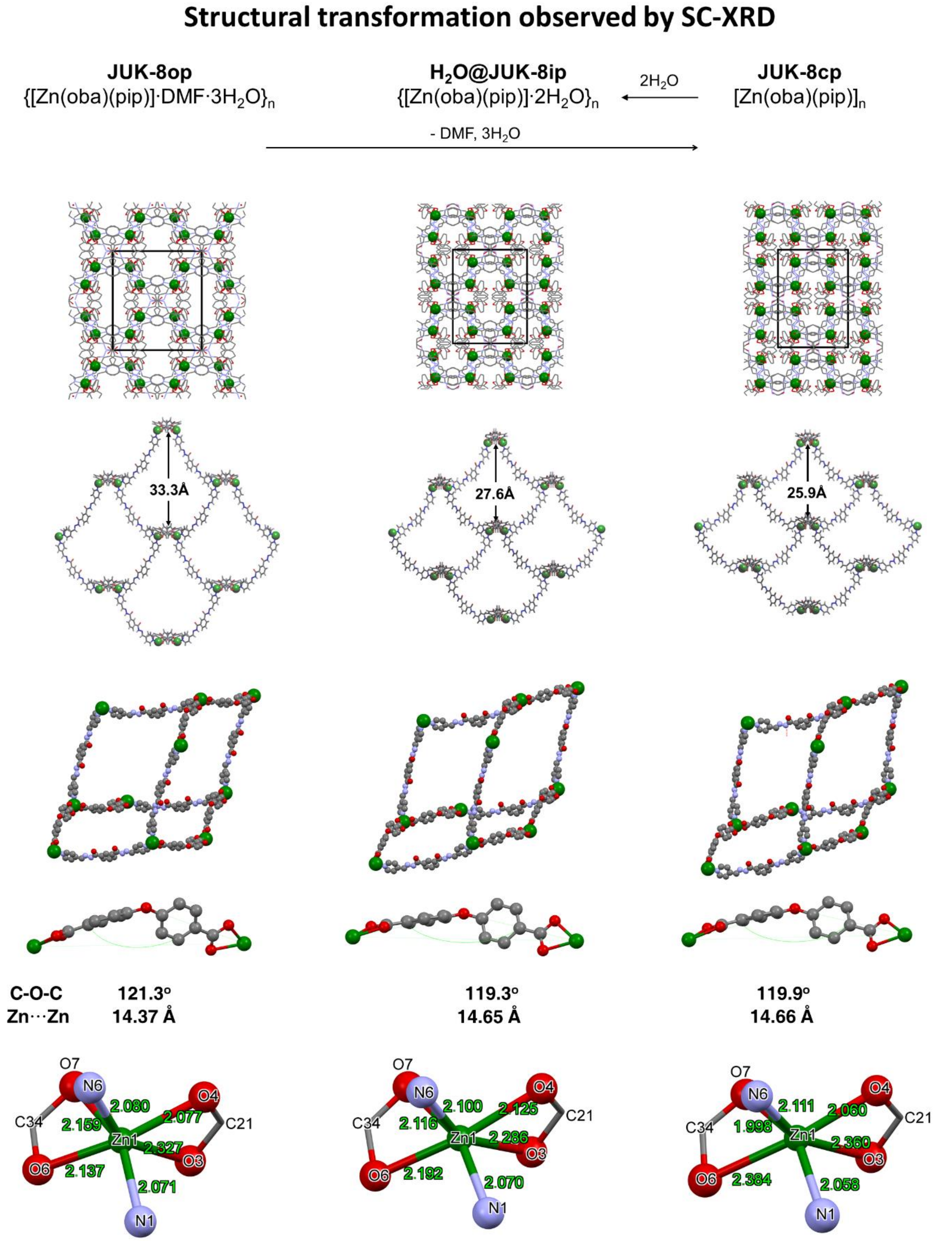

Figure S3 Comparison of three guest-dependent crystalline phases of **JUK-8** elucidated by SC-XRD: crystal structures of the as-made **JUK-8op**[1], **H_2O@JUK-8ip**[1] and **JUK-8cp** (Zn-green, C-grey, O-red, N -blue, H atoms are omitted).

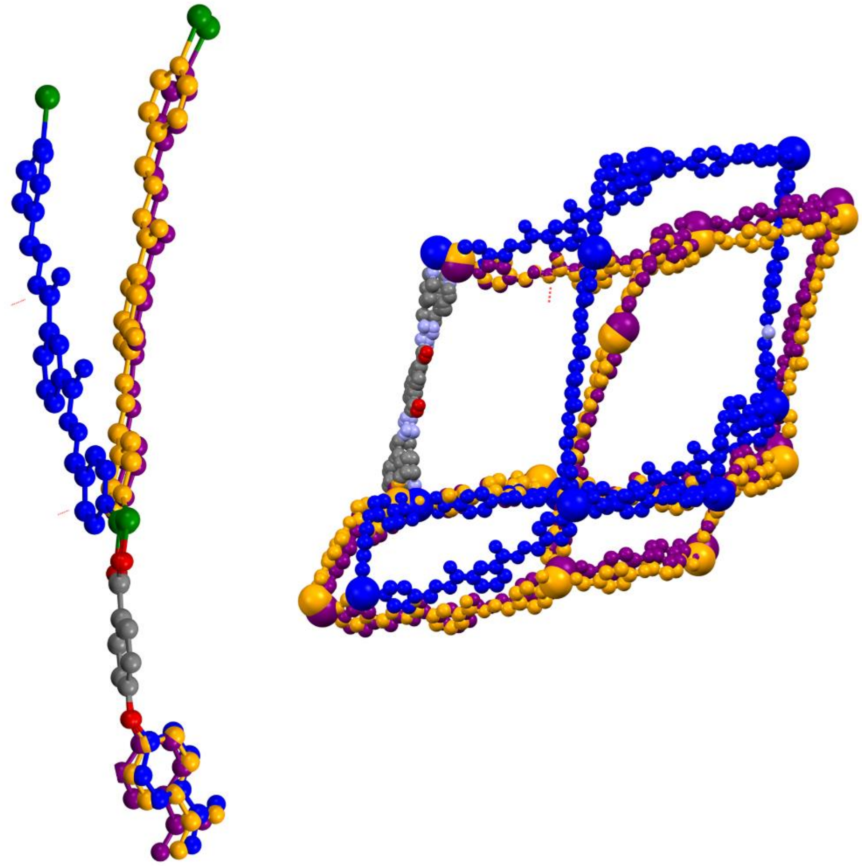

Figure S4 Partial overlapping of crystal structures of the as-synthesized **JUK-8op** (blue), **H_2O@JUK-8ip** (purple) and **JUK-8cp** (orange).

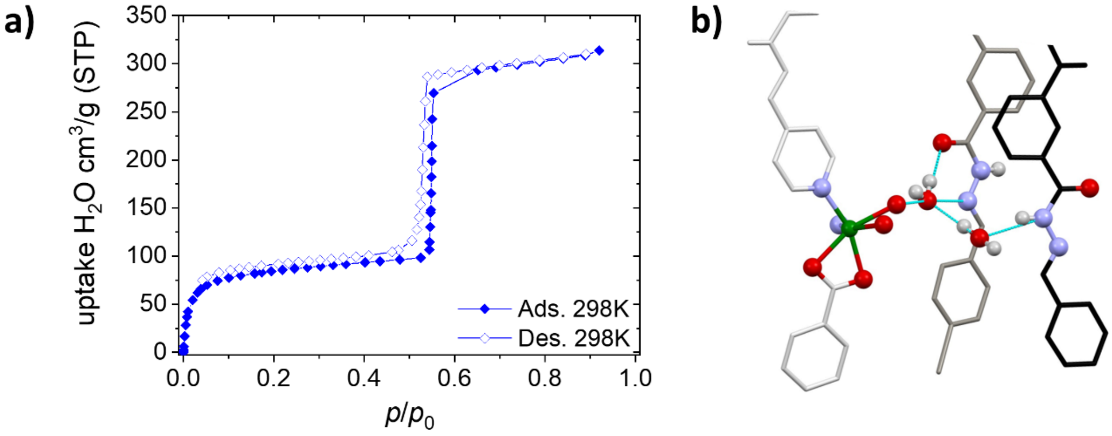

Figure S5 a) H_2O isotherm (298 K) for **JUK-8cp**. b) Hydrogen bonds (cyan lines) involving three independent subnetworks and water molecules[1].

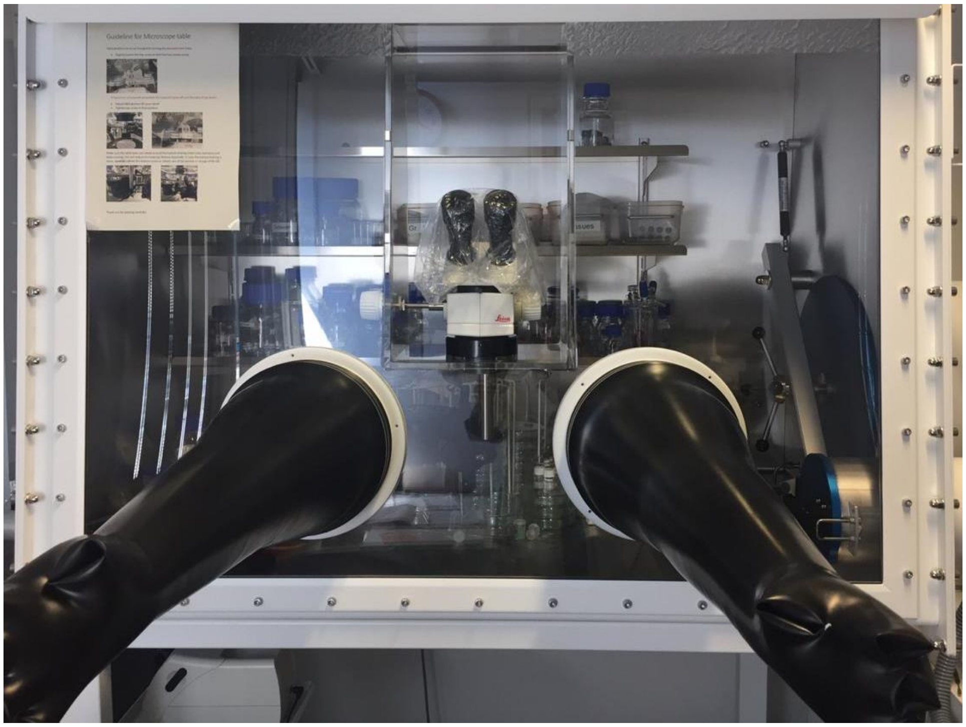

Figure S6 Glovebox with an inert atmosphere (Ar) equipped with the microscope.

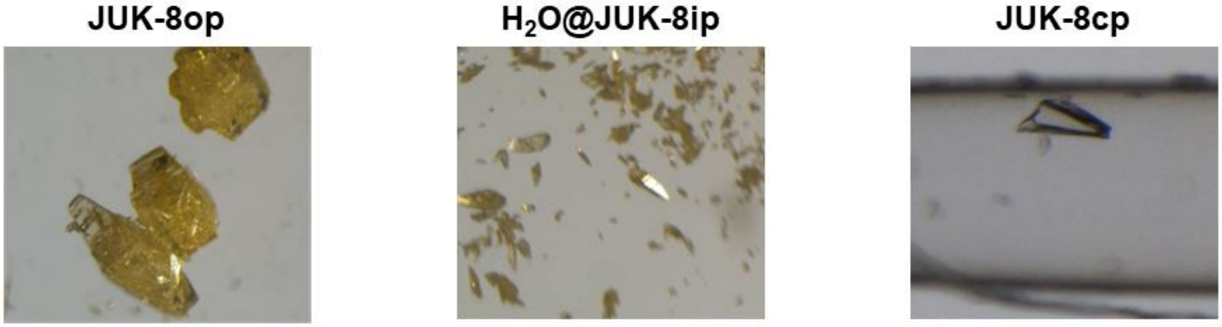

Figure S7 Optical microscopic images of **JUK-8op**, **H_2O@JUK-8ip** and **JUK-8cp** (closed in the borosilicate capillary).

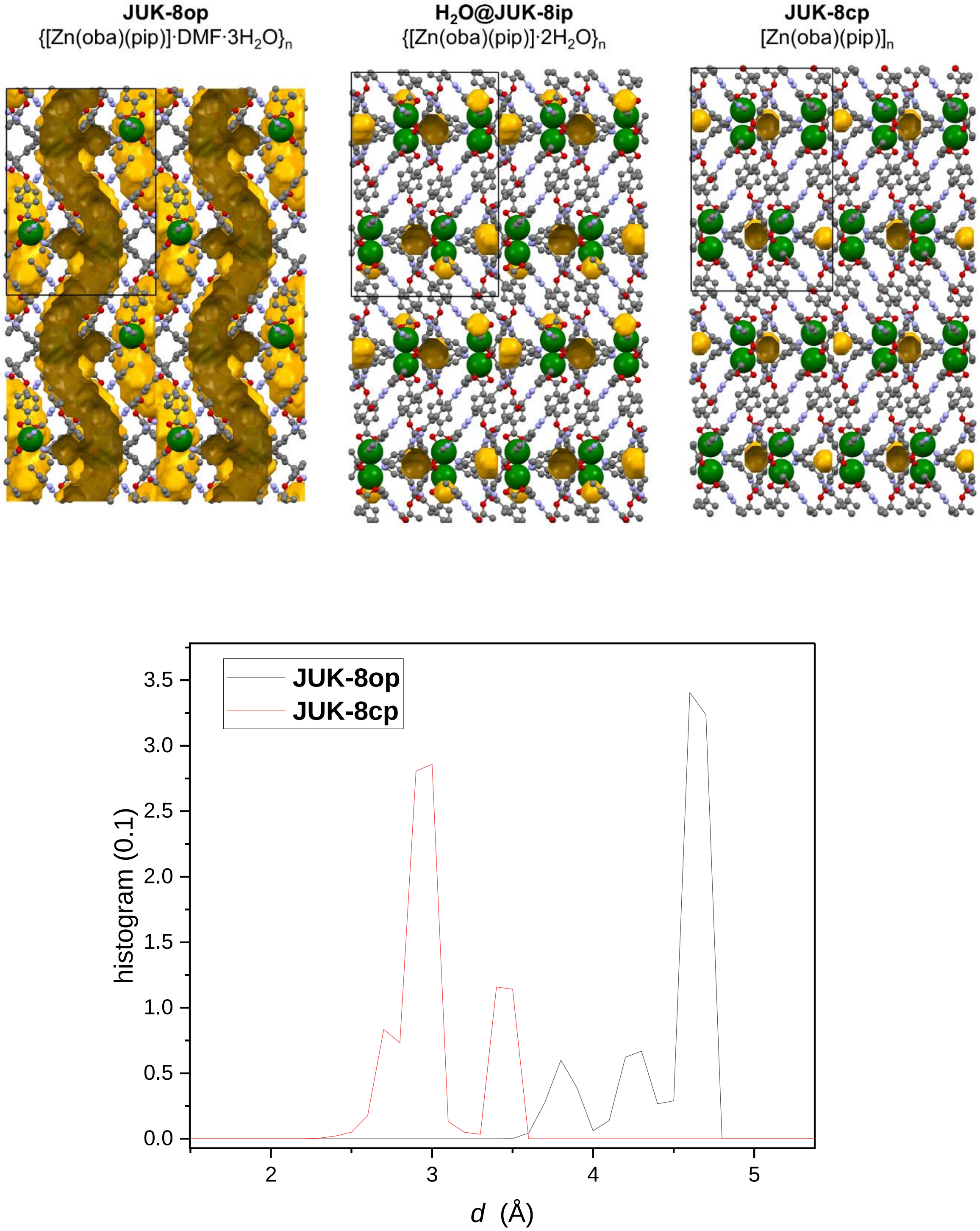

Figure S8 Structural transformation of **JUK-8** induced by desolvation: (Top) Voids calculated using the contact surface: **JUK-8op** (probe radius 1.75 Å)**;** $\mathbf{H_2O}$**@JUK-8ip** and **JUK-8cp** (probe radius 1.3 Å); view along the *a* axis. (Bottom) Pore size distribution calculated[2] using Zeo++ for **JUK-8cp** and **JUK-8op**.

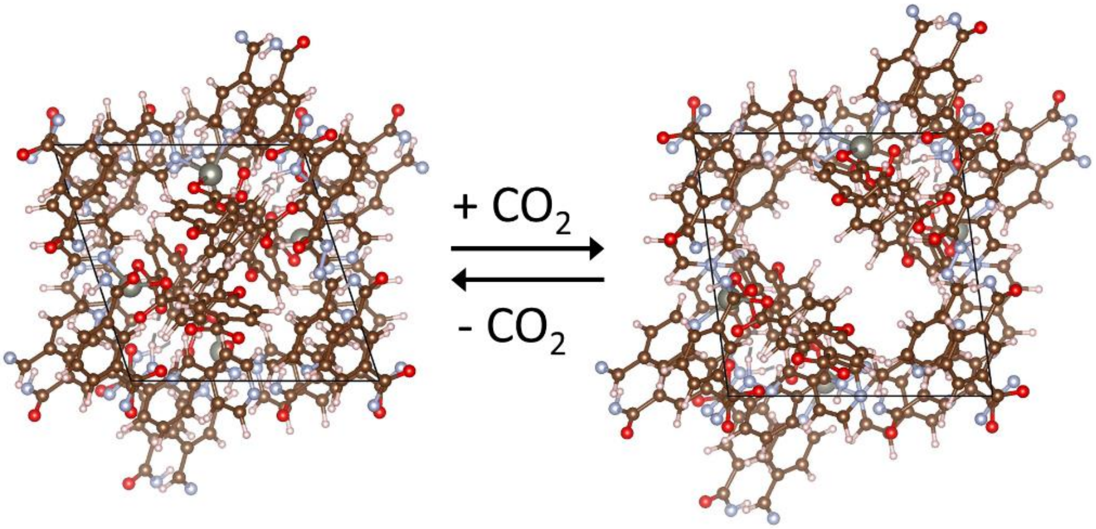

Figure S9 Structural transformation induced by CO_2 after exceeding gate opening pressure (p/p_0 = 0.08; 0.08 bar).

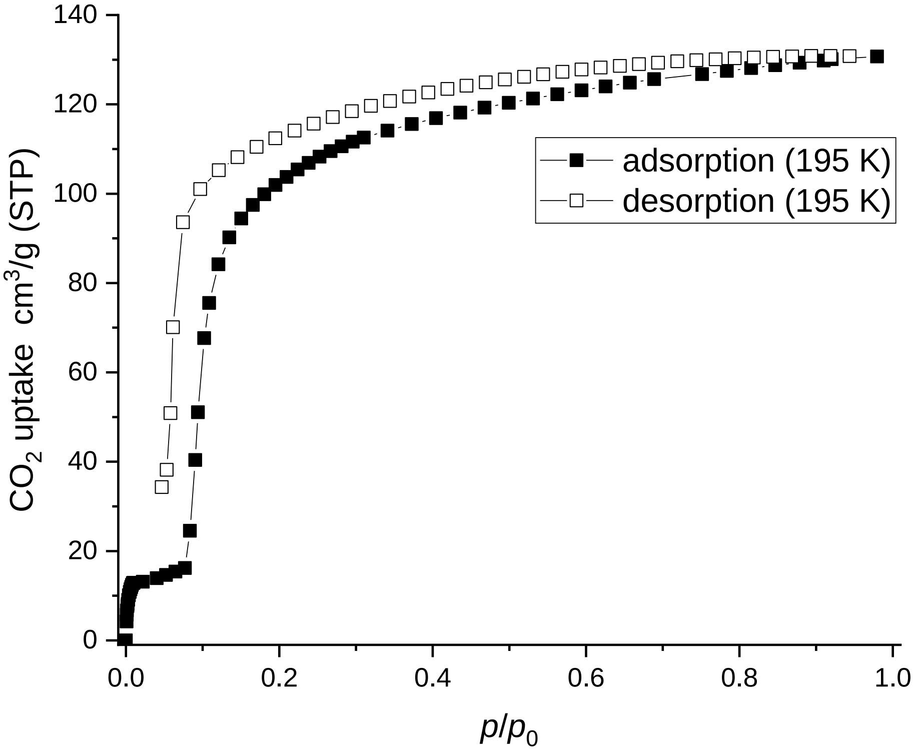

Figure S10 CO_2 isotherm (195 K) for a ground sample of **JUK-8**.

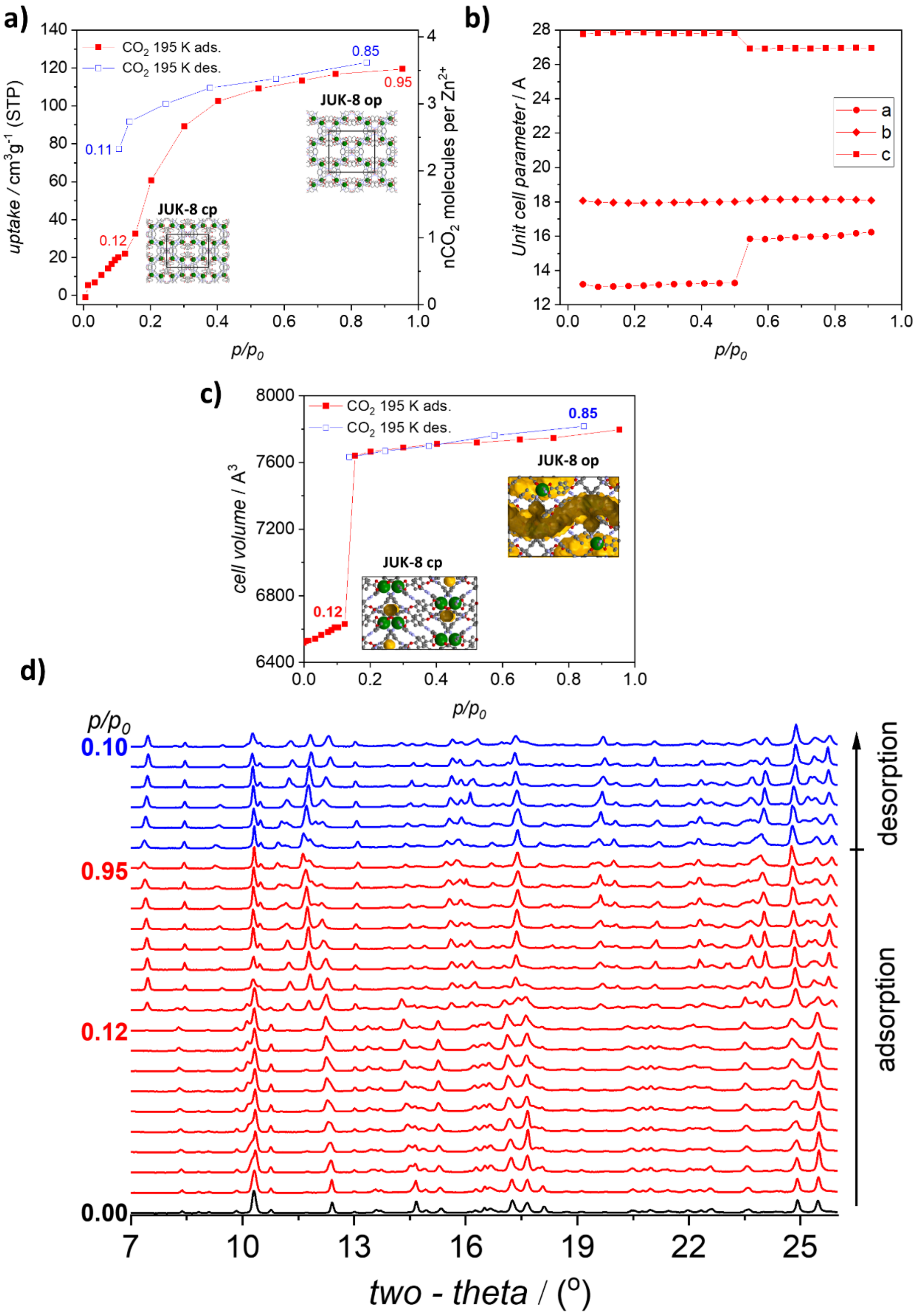

Figure S11 a) CO_2 (195 K) adsorption isotherm for **JUK-8cp**. b), c) Corresponding changes of unit cell parameters, and d) *in situ* variable pressure PXRD (λ = 1.54056 Å) during CO_2 adsorption and desorption. Full symbols - adsorption, open symbols – desorption.

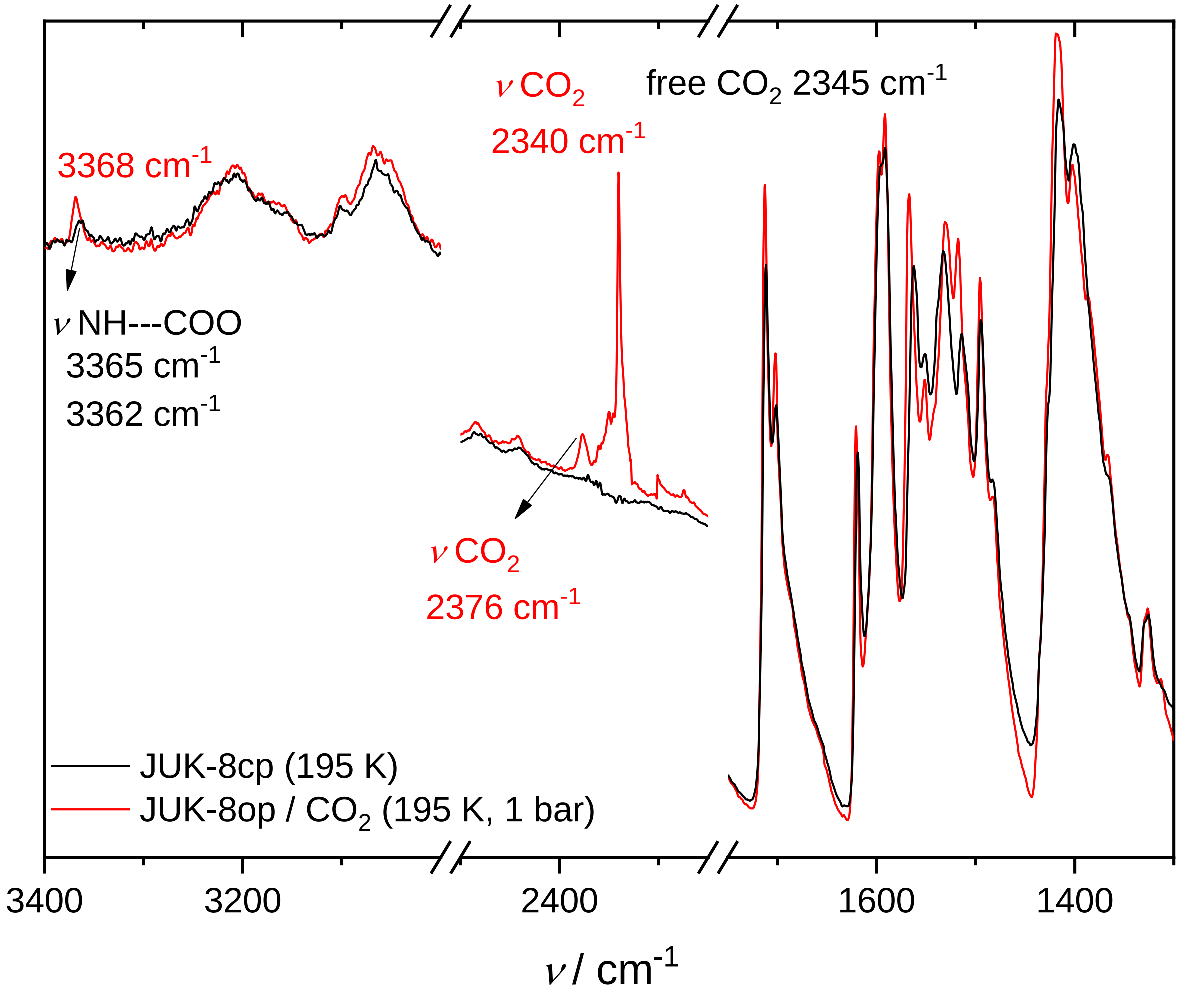

Figure S12 *In situ* IR spectra recorded during carbon dioxide adsorption. Black curve - **JUK-8cp** (195 K), red curve **-** CO_2 loaded **JUK-8op** (195 K; p/p_0 = 0.99).

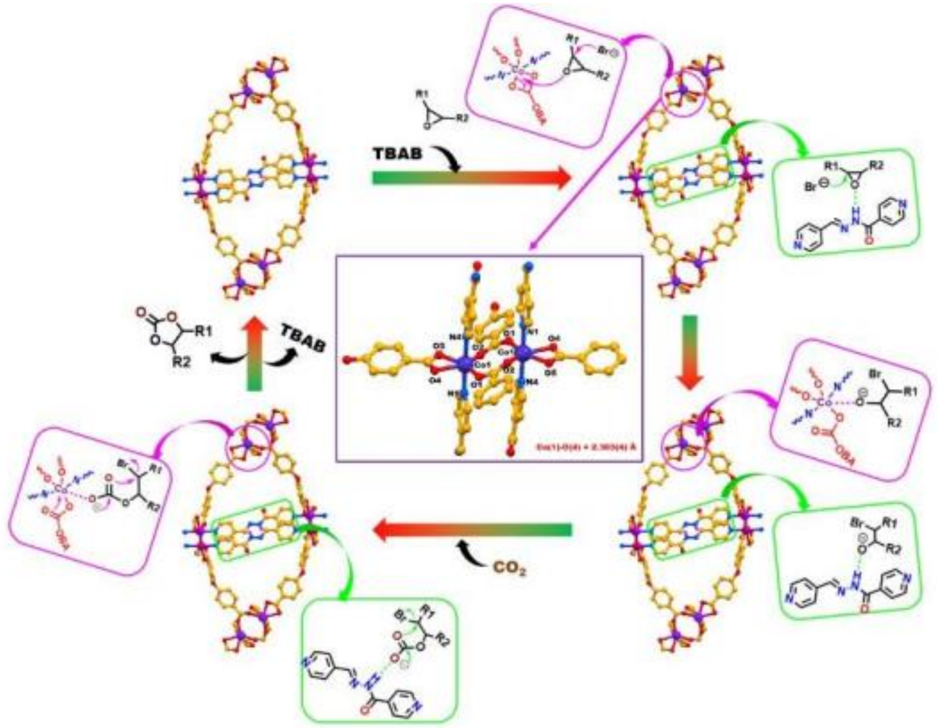

Figure S13 Proposed catalytic mechanism catalytic mechanism for CO_2 cycloaddition[3] that leads to terminal / internal epoxides using acylhydrazone MOF {[Co(OBA)(L)]·xG}n (**CoMOF-1**) (H_2OBA = 4,4′-Oxybis(benzoic acid); L = (E)-N'-(pyridin-4-ylmethylene) isonicotinohydrazide; G = DMF, EtOH, MeOH, H_2O) as heterogeneous catalyst. (Reproduced with permission from RSC)[3].

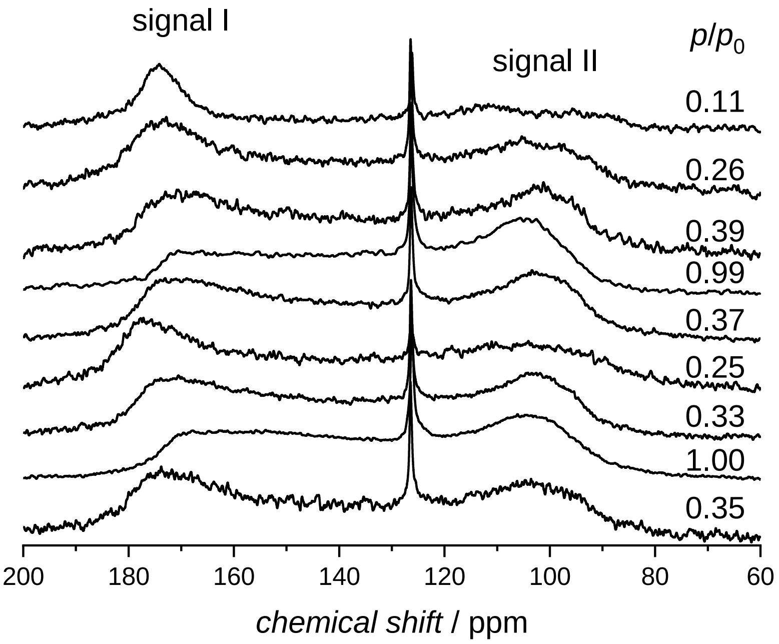

Figure S14 *In situ* $^{13}CO_2$ NMR cycling experiment.

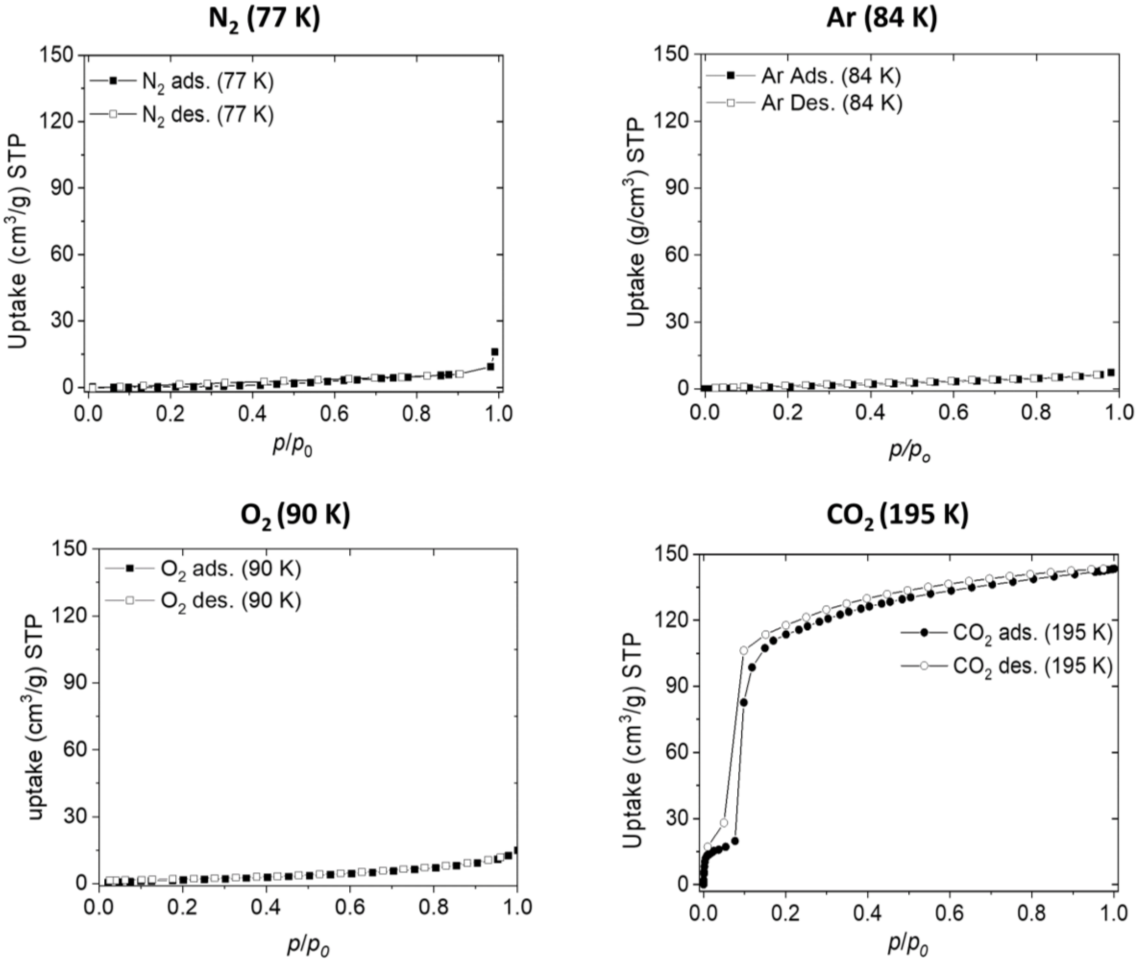

Figure S15 Adsorption properties of **JUK-8**: Single component isotherms for CO_2 (195 K), N_2 (77 K), O_2 (90K) and Ar (84 K).

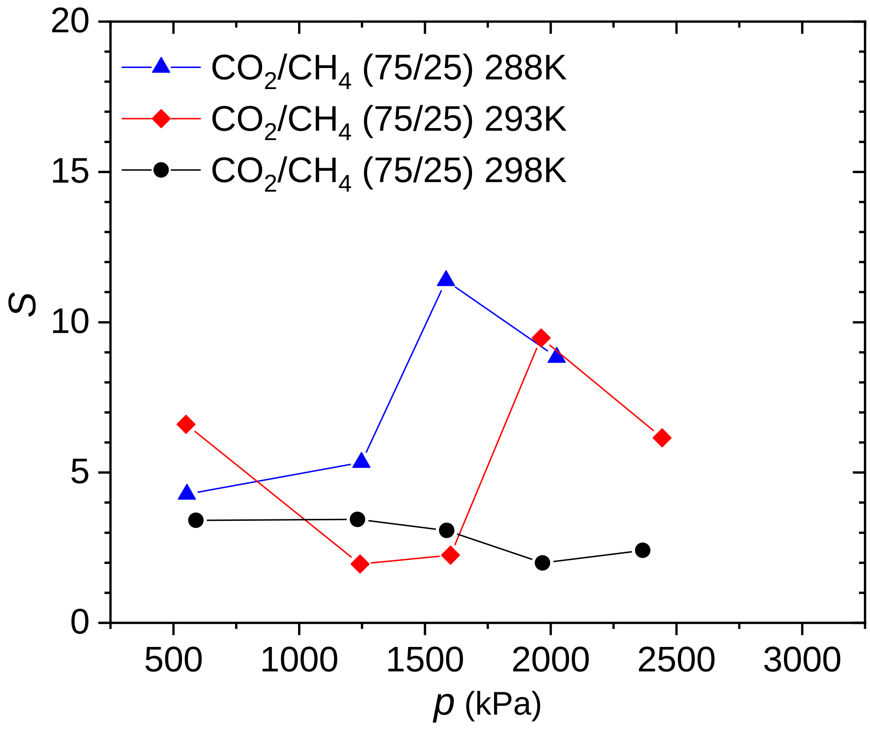

Figure S16 Pressure dependence of the selectivity factor *S* at 288 K, 293 K and 298 K.

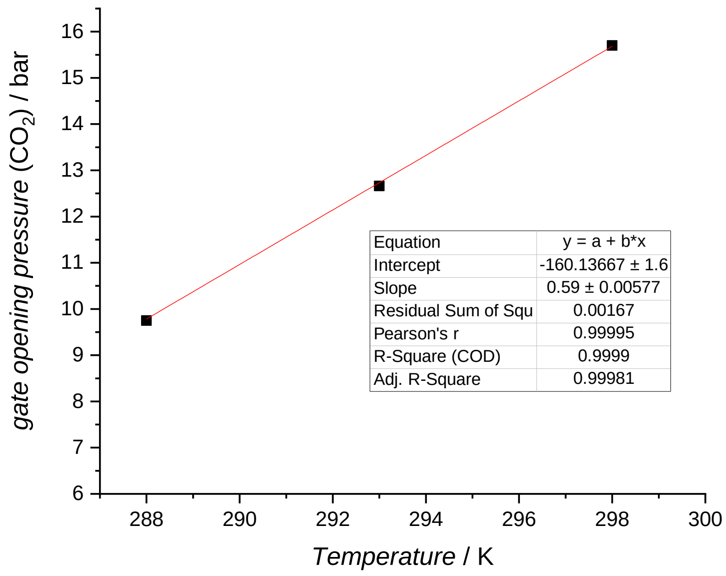

Figure S17 Correlation between CO_2 induced gate opening pressure (*gop*) and the temperature observed in the mixed gas adsorption. Gas mixture of 75 % CO_2 and 25 % CH_4 (*v/v*) at 298 K, 293 K and 288 K.

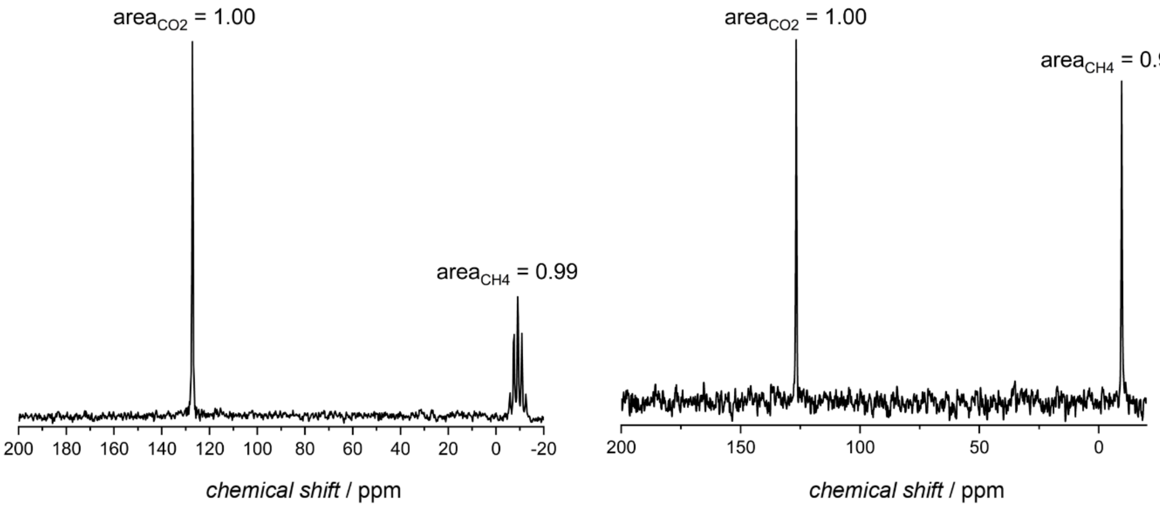

Figure S18 *In situ* ^{13}C NMR spectra of the pure of $^{13}CO_2/^{13}CH_4$ (1 : 1) gas mixture measured at 1.00 bar without (left) and with (right) ^{1}H decoupling. Note that the signal intensities (integral areas) for both measurements are very close to the expected 1 : 1 ratio.

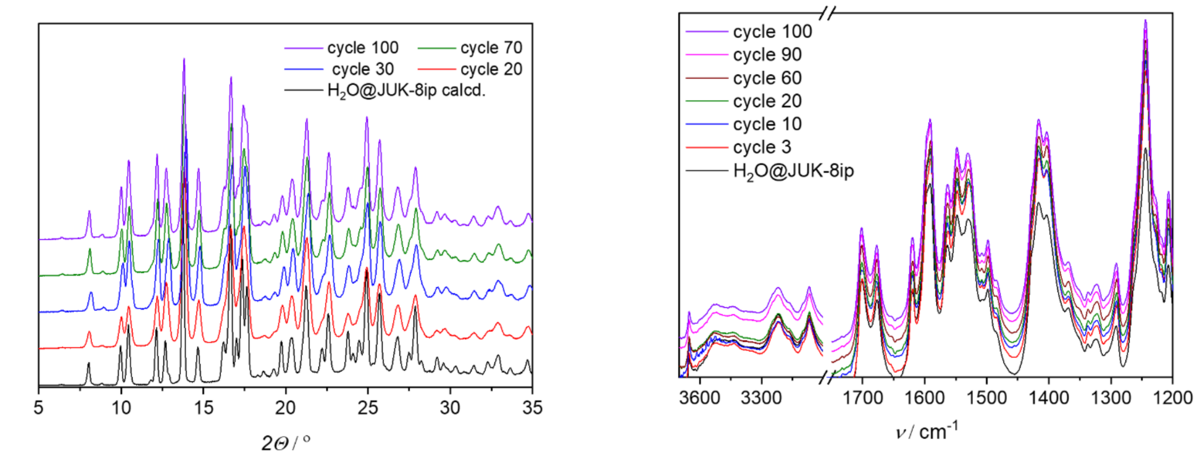

Figure S19 PXRD patterns (left) and IR spectra monitoring stability of **JUK-8** during the repeatable water solvation and desolvation processes.

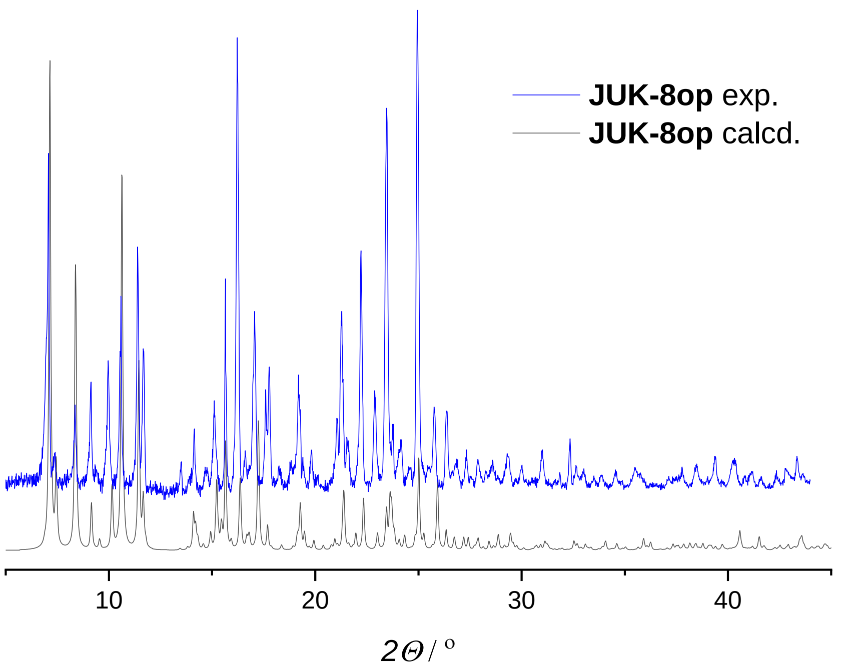

Figure S20 Comparison of PXRD patterns for **JUK-8op:** the calculated, based on SC-XRD measurement at 120 K (black) and the experimental, measured for the as-synthesized polycrystalline sample at 295 K (blue).

Tables

Table S1 Hydrogen bond parameters for **JUK-8op, H_2O@JUK-8ip** and **JUK-8cp** (bond lengths Å, and angles in °).

D—H···A	D—H	H···A	D···A	D—H···A
**JUK-8op**[1]				
N4—H4…O3^{i}	0.88	1.97	2.790(9)	156
**H_2O@JUK-8ip**[1]				
N4—H4…O3^{ii}	0.88	2.00	2.822 (3)	154
N3—H3…O2W	0.88	2.08	2.910 (5)	157
O1W—H1W1…O4^{iii}	0.89 (5)	2.03 (5)	2.787 (4)	142 (4)
O1W—H2W1…O2^{iv}	0.82 (5)	2.10 (5)	2.917 (4)	172 (5)
O2W—H1W2…O1W^{v}	0.84 (1)	1.88 (1)	2.720 (5)	174 (4)
**JUK-8cp**				
N4—H4…O3^{vi}	0.90(2)	1.92(2)	2.798(5)	165(5)
Symmetry codes: (i) x-3/2, -y+1/2, -z+1; (ii) -x-1/2, y-1/2, -z+1/2; (iii) x, -y+1, z-1/2; (iv) -x, -y, -z; (v) x-1, y, z; (vi) 3/2-x, 1/2-y, -z.				

Table S2 π···π interactions for **JUK-8op, H_2O@JUK-8ip** and **JUK-8cp** (bond lengths in Å, and angles in °).

		Distance Cg(I)…Cg(J)	Shift*
**JUK-8op**[1]			
Cg1…Cg2^{i}		3.869(5)	1.263
**H_2O@JUK-8ip**[1]			
Cg1…Cg2^{ii}		3.9019(17)	1.347
Cg2…Cg2^{iii}		3.8630(18)	1.893
**JUK-8cp**			
Cg1…Cg3^{i}		3.8531(9)	1.471
Cg3…Cg3^{ii}		3.7966(9)	1.802
	Cg1: N1 C1 C2 C3 C4 C5; Cg2: C8 C9 C10 C11 C12 C13<br>Symmetry codes: (i) 3/2-x,1/2-y,1-z, (ii) -1/2-x,1/2-y,-z, (iii) -1-x,-y,-,z<br>* distance between Cg(I) and Perpendicular Projection of Cg(J) on Ring I		

Table S3 Crystallographic data for **JUK-8op, H_2O@JUK-8ip** and **JUK-8cp**.

**Compound name**	**JUK-8op**	**H_2O@JUK-8ip**	**JUK-8cp**
References	[1]	[1]	This work
Chemical formula	$C_{34}H_{24}N_6O_7Zn$	$C_{34}H_{28}N_6O_9Zn$	$C_{34}H_{24}N_6O_7Zn$
Formula Mass	693.96	729.99	693.98
		**Crystal data**	
Crystal system, space group	Monoclinic, C2/c	Monoclinic, C2/c	Monoclinic, C2/c
Temperature (K)	120(2)	130(2)	100(2)
a, b, c (Å)	16.9782(13), 18.5080(14), 26.157(2)	13.8699(3), 17.5411(3), 27.7173(3)	12.980(3), 18.060(4), 27.940(6)
α, β, γ (°)	90, 101.653(8), 90	90, 94.007(1), 90	90, 94.34(3), 90
Unit cell volume/Å^3	8050.1(11)	6726.9(2)	6531(3)
Z	8	8	8
Crystal size (mm)	0.30×0.20×0.20	0.40×0.25×0.20	-
Diffractometer	SuperNova, Dual, Cu at zero, Atlas		BESSY MX BL -14.3
Radiation type	MoKα		Synchrotron (λ = 0.89390 Å)
Absorption correction type	Multi-scan		Not given
Absorption coefficient, μ/mm^{-1}	0.66	0.79	1.488
No. of reflections measured/independent	14164/7442	41158/8938	16757/4727
R_{int}	0.1199	0.0562	0.0742
θ range/°	3.355-28.476	2.750-29.697	1.839- 31.684
**Refinement**			
No. of data in refinement / with $F^2>2\sigma(F^2)$	7442/4712	8938/5823	4727/3658
No. of parameters/restrains	432/0	463/3	439/2
Final R_1 value (I > 2σ(I))	0.1193	0.059	0.0557
Final wR(F^2) values (I > 2σ(I))	0.3288	0.128	0.1456
Goodness of fit on F^2	1.032	1.085	1.099

Table S4 Selected bond lengths (Å) and angles (°) for **JUK-8op, H_2O@JUK-8ip** and **JUK-8cp**.

	**JUK-8op**[1]	**H_2O@JUK-8ip**[1]	**JUK-8cp**
Zn1–N1	2.071(7)	2.070(3)	2.058(4)
Zn1–N6	2.079(8)	2.099(3)	2.111(4)
Zn1–O3	2.327(6)	2.286(2)	2.360(3)
Zn1–O4	2.078(5)	2.125(2)	2.060(4)
Zn1–O6	2.137(9)	2.193(2)	2.383(4)
Zn1–O7	2.159(7)	2.116(2)	1.999(4)
N1–Zn1–O4	103.5(2)	98.26(10)	101.27(16)
N1–Zn1-N6	102.9(3)	96.57(10)	96.11(17)
O4–Zn1–N6	102.9(3)	94.04(9)	98.16(15)
N1–Zn1–O6	95.2(3)	97.55(10)	93.55(15)
O4–Zn1–O6	153.6(3)	159.79(9)	159.23(14)
N6–Zn1–O6	100.6(3)	96.56(9)	94.60(14)
N1–Zn1–O7	152.1(3)	158.10(10)	151.30(18)
O4–Zn1–O7	98.5(3)	101.42(8)	103.40(16)
N6–Zn1–O7	92.6(3)	91.57(10)	94.69(18)
N1–Zn1–O3	91.0(2)	92.33(9)	89.15(14)
N6–Zn1–O3	151.8(3)	153.07(8)	157.50(14)
O6–Zn1–O3	102.4(2)	107.42(8)	106.93(12)
O7–Zn1–O3	85.6(2)	89.46(8)	90.95(14)

Table S5 Selectivity factor (**S**) of CO_2/CH_4 for various MOFs, zeolites and activated carbon.

Material	S of CO_2/CH_4	Temperature	Reference
**JUK-8**	8.83 (20 bar), 5.33 (12 bar) 4.27 (6 bar)	288 K	This work
	9.48 (20 bar), 2.25 (12 bar), 6.6 (6 bar)	293 K	
	1.99 (20 bar), 3.07 (12 bar), 3.4 (6 bar)	298 K	
**Cu(H-pymo)$_2$**	1.57 (28 bar)	273 K	[4]
**Zn$_2$(bttb)**	1.47 (17.5 bar)	298 K	[5]
**MIL-125(Ti)**	4.4 (9.8 bar), 6.0 (1 bar)	298 K	[6]
	3.1 (9.8 bar), 5.1 (1 bar)	273 K	
**MIX-MIL-125(Ti)**	2.9 (9.8bar)<br>4.2 (1 bar)	298 K	[6]
	2.6 (9.8 bar)<br>4.6 (1 bar)	273 K	
**NH$_2$-MIL-125(Ti)**	2.5 (9.8 bar), 3.9 (1 bar)	298 K	[6]
	2.2 (9.8 bar), 4.1 (1 bar)	273 K	
**ZIF 68**	3.8 (1bar)	298 K	[7]
	5.0 (1 bar)	273 K	
**Zeolite 13X**	3.6 (1 bar)	298 K	[8]
**Zeolite 13X (UOP)**	2.2 (9.8 bar), 5.9 (1 bar)	298 K	[6]
	1.9 (9.8 bar), 4.6 (1 bar)	273 K	
**ZIF-69**	3.4 (1bar)	298 K	[7]
	5.1 (1 bar)	273 K	
**ZIF-70**	3.2 (1bar)	298 K	[7]
	5.2 (1 bar)	273 K	
**Activated carbon (ChemFFX)**	2.3 (1 bar) , 1.92 (9.8 bar)	298 K	[6]
	2.2 (1 bar) , 1.9 (9.8 bar)	273 K	
**Activated carbon, A35/4,**	2.2 (1 bar) , 3.39 (9.8 bar)	298	[9]
**[H$_3$O][Zn$_7$(μ_3-OH)$_3$(bbs)$_6$]**	3.5 (1 bar)	298 K	[10]
**Mn(ndc)**	1.87 (1 bar)	298 K	[11]

References

(1) Roztocki, K.; Formalik, F.; Krawczuk, A.; Senkovska, I.; Kuchta, B.; Kaskel, S.; Matoga, D. Collective Breathing in an Eightfold Interpenetrated Metal–Organic Framework: From Mechanistic Understanding towards Threshold Sensing Architectures. *Angewandte Chemie International Edition* **2020**, *59* (11), 4491–4497. https://doi.org/10.1002/anie.201914198.

(2) Willems, T. F.; Rycroft, C. H.; Kazi, M.; Meza, J. C.; Haranczyk, M. Algorithms and Tools for High-Throughput Geometry-Based Analysis of Crystalline Porous Materials. *Microporous and Mesoporous Materials* **2012**, *149* (1), 134–141. https://doi.org/10.1016/j.micromeso.2011.08.020.

(3) Parmar, B.; Patel, P.; Pillai, R. S.; Kureshy, R. I.; Khan, N. H.; Suresh, E. Efficient Catalytic Conversion of Terminal/Internal Epoxides to Cyclic Carbonates by Porous Co(Ii) MOF under Ambient Conditions: Structure–Property Correlation and Computational Studies. *J. Mater. Chem. A* **2019**, *7* (6), 2884–2894. https://doi.org/10.1039/C8TA10631B.

(4) Galli, S.; Masciocchi, N.; Tagliabue, G.; Sironi, A.; Navarro, J. A. R.; Salas, J. M.; Mendez-Liñan, L.; Domingo, M.; Perez-Mendoza, M.; Barea, E. Polymorphic Coordination Networks Responsive to CO2, Moisture, and Thermal Stimuli: Porous Cobalt(II) and Zinc(II) Fluoropyrimidinolates. *Chemistry – A European Journal* **2008**, *14* (32), 9890–9901. https://doi.org/10.1002/chem.200801048.

(5) Bae, Y.-S.; Farha, O. K.; Hupp, J. T.; Snurr, R. Q. Enhancement of CO2/N2 Selectivity in a Metal-Organic Framework by Cavity Modification. *J. Mater. Chem.* **2009**, *19* (15), 2131–2134. https://doi.org/10.1039/B900390H.

(6) Rada, Z. H.; Abid, H. R.; Shang, J.; He, Y.; Webley, P.; Liu, S.; Sun, H.; Wang, S. Effects of Amino Functionality on Uptake of CO2, CH4 and Selectivity of CO2/CH4 on Titanium Based MOFs. *Fuel* **2015**, *160*, 318–327. https://doi.org/10.1016/j.fuel.2015.07.088.

(7) Phan, A.; Doonan, C. J.; Uribe-Romo, F. J.; Knobler, C. B.; O'Keeffe, M.; Yaghi, O. M. Synthesis, Structure, and Carbon Dioxide Capture Properties of Zeolitic Imidazolate Frameworks. *Acc. Chem. Res.* **2010**, *43* (1), 58–67. https://doi.org/10.1021/ar900116g.

(8) Liang, Z.; Marshall, M.; Chaffee, A. L. CO2 Adsorption-Based Separation by Metal Organic Framework (Cu-BTC) versus Zeolite (13X). *Energy Fuels* **2009**, *23* (5), 2785–2789. https://doi.org/10.1021/ef800938e.

(9) Heuchel, M.; Davies, G. M.; Buss, E.; Seaton, N. A. Adsorption of Carbon Dioxide and Methane and Their Mixtures on an Activated Carbon: Simulation and Experiment. *Langmuir* **1999**, *15* (25), 8695–8705. https://doi.org/10.1021/la9904298.

(10) Neofotistou, E.; D. Malliakas, C.; N. Trikalitis, P. Unprecedented Sulfone-Functionalized Metal–Organic Frameworks and Gas-Sorption Properties. *Chemistry – A European Journal* **2009**, *15* (18), 4523–4527. https://doi.org/10.1002/chem.200900341.

(11) Moon, H. R.; Kobayashi, N.; Suh, M. P. Porous Metal−Organic Framework with Coordinatively Unsaturated MnII Sites:Sorption Properties for Various Gases. *Inorg. Chem.* **2006**, *45* (21), 8672–8676. https://doi.org/10.1021/ic0611948.
